# Alkylated DNA repair by a novel HhH-GPD family protein from Crenarchaea

**DOI:** 10.1093/nar/gkaf012

**Published:** 2025-01-22

**Authors:** Likui Zhang, Tian Gao, Zheng Li, Cai Chen, Donghao Jiang, Youcheng Yin, Yaqi Zheng, Peng Cao, Yong Gong, Zhihui Yang

**Affiliations:** College of Environmental Science and Engineering, Yangzhou University, No. 196 Huayang West Road, Yangzhou City, 225127, China; College of Environmental Science and Engineering, Yangzhou University, No. 196 Huayang West Road, Yangzhou City, 225127, China; College of Plant Protection, Agricultural University of Hebei, No. 2596 Lekai South Street, Baoding City, Lianchi District, Hebei Province 071001, China; College of Environmental Science and Engineering, Yangzhou University, No. 196 Huayang West Road, Yangzhou City, 225127, China; College of Environmental Science and Engineering, Yangzhou University, No. 196 Huayang West Road, Yangzhou City, 225127, China; College of Environmental Science and Engineering, Yangzhou University, No. 196 Huayang West Road, Yangzhou City, 225127, China; College of Environmental Science and Engineering, Yangzhou University, No. 196 Huayang West Road, Yangzhou City, 225127, China; College of Chemistry and Life Science, Beijing University of Technology, No. 100 Pingleyuan Road, Chaoyang District, Beijing 100124, China; Beijing Synchrotron Radiation Facility, Institute of High Energy Physics, Chinese Academy of Sciences, No. 19 Yuquan Road, Shijingshan District, Beijing 100040, China; College of Plant Protection, Agricultural University of Hebei, No. 2596 Lekai South Street, Baoding City, Lianchi District, Hebei Province 071001, China

## Abstract

HhH-GPD (helix–hairpin–helix-glycine/proline/aspartate) family proteins are involved in DNA damage repair. Currently, mechanism of alkylated DNA repair in Crenarchaea has not been fully clarified. The hyperthermophilic model crenarchaeon *Saccharolobus islandicus* REY15A possesses a novel HhH-GPD family protein (Sis-HhH-GPD), where its Ser152 corresponds to a conserved catalytic Asp in other HhH-GPD homologs. Herein, we report that Sis-HhH-GPD is a novel bi-functional glycosylase, capable of removing both 1-methyladenine (1-meA) from DNA and alkylated bases from DNA created by methyl methanesulfonate (MMS). Mutational analyses show that E134 is essential for catalysis, whereas S152 is not essential. Sis-HhH-GPD might utilize aromatic rings of Y154 and W57 to stack against 1-meA base for flipping-out and then be removed by E134. Additionally, R157, R161 and R200 participate in catalysis. Among four cysteine residues that potentially coordinate with the Fe-S cluster loop, C203, C210 and C219 are involved in catalysis. Importantly, Sis-HhH-GPD is responsible for repair of alkylated DNA created by MMS *in vivo*. Interestingly, genetic complementary data have confirmed physiological function of Sis-HhH-GPD in alkylated DNA repair and clarified functional roles of its four cysteine residues *in vivo*. Overall, we provide first evidence that HhH-GPD family protein from Crenarchaea functions in alkylated DNA repair.

## Graphical Abstract

**Graphical Abstract:**
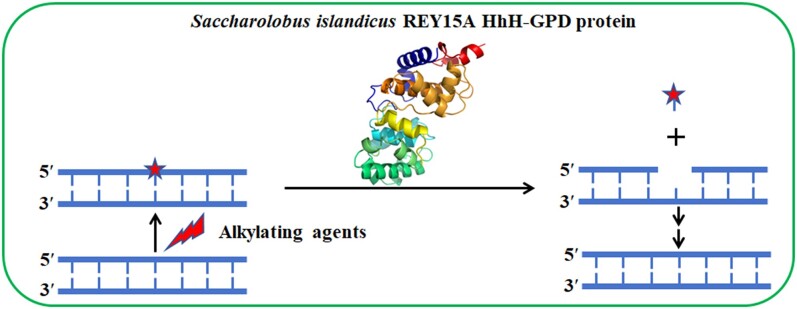

## Introduction

Thousands of DNA base lesions are created in cells upon exposure to endogenous and exogenous DNA damaging agents, among which aberrantly methylated bases account for a large proportion (1). Several alkylated bases have been identified to date (2–4), including N^7^-methylguanine (7-meG), 3-methyladenine (3-meA) and O^6^-methylguanine (O^6^-meG), 1-methyladenine (1-meA), 3-methylcytosine (3-meC), O^4^-methylthymine (O^4^-meT) and methyl phosphotriester (MPT) (5). 7-meG, 3-meA and O^6^-meG represent major alkylated bases in DNA, while 1-meA, 3-meC, O^4^-meT and MPT occur in DNA in relatively small amounts (6). It has been estimated that there are approximate 6 000 7-meG and 1 200 3-meA lesions per mammalian cell per day (7). O^6^-meG and O^4^-meT are highly mutagenic and genotoxic, whereas 3-meA, 1-meA, 3-meC and 3-meG are cytotoxic rather than mutagenic since they can block DNA replication and transcription (5,6). Thus, alkylated bases in DNA need to be quickly repaired to maintain genomic stability once created in cells.

Generally, alkylation lesions in DNA are repaired by direct repair (DR), base excision repair (BER), nucleotide excision repair (NER) and other pathways (4,8,9), which is dependent on alkylated base types. In an example of O^6^-meG, which is usually generated in DNA after exposed to alkylating agents, such as methyl methanesulfonate (MMS), O^6^-meG can be repaired by DR via transferring a methyl group to a specific cysteine residue of a methyltransferase itself (9,10). On the other hand, abundant and cytotoxic 3-meA bases are repaired by BER, which is initiated by 3-alkyladenine DNA glycosylase II (AlkA) (11). In addition to these two major pathways, *Escherichia coli* encodes an oxidative DNA demethylase, which is named AlkB, capable of repairing cytotoxic 1-meA and 3-meC lesions in DNA (12–14). Interestingly, eukaryotic cells possess a homolog of *E. coli* AlkB protein. These eukaryotic AlkB homologs can excise 1-meA and 3-meC from both DNA and RNA, thereby expanding alkylation repair pathway diversities (15–17). Last, Plosky *et al.* proposed that NER pathway is responsible for repair of 7-meG and 3-meA in DNA (18).

BER, which is present in all three kingdoms of life, is a typical repair pathway for removal of DNA damaged bases that potentially cause minor DNA conformational change, including alkylated bases (19). In general, BER is initiated by DNA glycosylase. According to their structural and functional homology, DNA glycosylases have been classified into four superfamilies: uracil DNA glycosylase, endonuclease VIII-like glycosylase, 3-methyl-purine glycosylase and helix–hairpin–helix (HhH) DNA glycosylase (20). Each DNA glycosylase superfamily contains the corresponding proteins that can typically excise a single damaged lesion from DNA. In addition, DNA glycosylases are divided into two functional categories: mono- and bi-functional enzymes (21). Mono-functional glycosylases possess only DNA glycosylase activity that can excise a damaged base from DNA to yield an AP (apurinic/apyrimidinic) site. In contrast, bi-functional enzymes can not only remove a damaged DNA base from DNA via their glycosylase activities, but also further cleave a backbone of the generated AP site by their AP lyase activities (22).

All reported HhH DNA glycosylases possess a common core structure that is composed of a signature HhH motif and a glycine/proline-rich loop followed by a conserved aspartate residue (GPD) (23,24). HhH-GPD DNA glycosylases include A/G-mismatch-specific adenine glycosylase (MutY), endonuclease III (EndoIII), 8-oxoguanine DNA glycosylase 1 (OGG1), 8-oxoguanine DNA glycosylase 2 (OGG2), N-methyl-purine-DNA glycosylase II (MpgII), alkyladenine DNA glycosylase and methyl-binding domain protein 4 (25). HhH-GPD DNA glycosylases are widespread in Bacteria, Archaea and Eukarya, capable of recognizing and excising a wide variety of damaged DNA lesions that derive from alkylation, oxidation and hydrolytic deamination (26,27). Generally, HhH-GPD DNA glycosylases excise a damaged base from DNA via flipping-out mechanism, by which this unwanted base is flipped out of DNA helix into an active site pocket of the enzyme and then cleaved (28). In OGGs and EndoIII, a conserved lysine residue that is positioned within an HhH-GPD motif serves as a catalytic nucleophile and a conserved aspartate that is located in the GPD loop is essential for catalytic activity (28). In addition, several HhH-GPD DNA glycosylases possess a Fe-S cluster loop, which is structurally coordinated with four conserved cysteine residues at their C-terminus. This Fe-S cluster loop has been shown to play a structural role in binding DNA (29,30).

Due to their living at high temperature, hyperthermophilic Archaea (HA) are facing a high risk of causing DNA damage. Currently, alkylated DNA repair pathways in HA have not been fully clarified. Recently, we have reviewed archaeal DNA alkylation repair mediated by DNA glycosylase and methyltransferase (31). These archaeal O^6^-methylguanine methyltransferase (MGMT) and AlkA are important enzymes used for alkylated DNA repair. Like bacteria and eukaryotes, Archaea have at least one MGMT homolog that is potentially involved in O^6^-meG repair. However, AlkA is only found in the limited archaeal phyla, especially in Euryarchaea. To date, only two AlkA proteins have been reported from the hyperthermophilic Euryarchaea: *Archaeoglobus fulgidus* and *Thermococcus gammatolerans*, capable of removing 1-meA from DNA (32–35), suggesting that AlkA might be involved in repair of 1-meA in DNA in Euryarchaea. No AlkB or AlkA homolog has been identified in Crenarchaea to date. Hence, a question arises: how Crenarchaea repair alkylated DNA except for O^6^-meG-containing DNA that can be repaired by crenarchaeal MGMT.

The hyperthermophilic crenarchaeon *Saccharolobus islandicus* REY15A is one of the most extensively studied model organisms used for archaeal DNA replication and repair (36,37). The genome of *S. islandicus* REY15A possesses the *SiRe_0278* gene, which encodes an HhH-GPD family protein (Sis-HhH-GPD) (38). In this work, we used a combination of biochemical and genetic methods to characterize Sis-HhH-GPD *in vitro* and *in vivo*, demonstrating that this protein is a bi-functional alkylated DNA glycosylase. Besides, we also revealed the roles of key amino acid residues of Sis-HhH-GPD in removal of alkylated bases from DNA. Notably, we confirmed that Sis-HhH-GPD is responsible for repair of alkylated DNA created by MMS *in vivo*.

## Materials and methods

### DNA substrates

Normal and damaged Hex-labeled oligonucleotides were synthesized by Sangon Biotech Company, China. The oligonucleotides were 5′-Hex-CGA ACT GCC TGG AAT CCT GAC GAC XTG TAG CGA ACG ATC ACC TCA-3′, where X is A, I (inosine), 1-meA, 8-oxoG (7,8-dihydro-8-oxoguanine), Tg (thymine glycol) or U (uracil). The Hex-labeled oligonucleotide duplexes were prepared by annealing the Hex-labeled oligonucleotides with their complementary strands. The annealing reactions were performed in annealing buffer (20 mM Tris-Cl, pH 8.0, and 100 mM NaCl) at 100°C for 3 min, and then cooled down slowly to room temperature.

### Sequence alignment and phylogenetic analysis

We retrieved sequences of Sis-HhH-GPD and other HhH-GPD DNA glycosylases from NCBI and selected the conserved HhH-GPD and Fe-S cluster loop sequences, and then submitted them to the website (https://www.genome.jp/tools-bin/clustalw) for alignment. And then the aligned sequences were submitted into the website (https://espript.ibcp.fr/ESPript/cgi-bin/ESPript.cgi) for making a sequence alignment figure. Using MEGA-X software, we submitted the sequences of Sis-HhH-GPD and other HhH-GPD homologs to construct a maximum likelihood unrooted phylogenetic tree. Bootstrapping was performed with 1000 replicates.

### Cloning, expression and purification

Using the genomic DNA of *S. islandicus* REY15A as a template, we used the Phusion DNA polymerase (Thermo Scientific, Waltham, MA, USA) to amplify the *SiRe_0278* gene with the forward and reverse primers harboring *Bam*HI and *Xho*I restriction sites, respectively. These primer sequences are summarized in Table S1. The polymerase chain reaction (PCR) reaction was performed by the following thermal program: initial heating at 95°C for 3 min, followed by 30 cycles of 95°C for 30 s, 55°C for 30 s and 72°C for 1 min, and a final extension step at 72°C for 5 min. The amplified DNA product was digested with *Bam*HI/*Xho*I and then inserted into the pET-28a_Plus vector. The resulting recombinant plasmid containing the *SiRe_0278* gene was verified by sequencing and then transformed by heating shock at 42°C for 90 s into the *E. coli* BL21 (DE3) pLysS cells (Transgen, Beijing, China) for protein expression. The expression strain *E. coli* harboring the *SiRe_0278* gene was grown in the LB (Luria-Bertani) medium with 17 μg/ml chloramphenicol and 50 μg/ml kanamycin. The isopropyl thiogalactoside (IPTG) at a final concentration of 0.1 mM was added to the culture when the OD_600_ value of the culture reached 0.6. The culture was further shaken for about 10 h at room temperature.

The cultured cells were collected by centrifugation and resuspended in Ni column buffer A containing 20 mM Tris-HCl (pH 8.0), 1 mM dithiothreitol (DTT), 500 mM NaCl, 50 mM imidazole and 10% glycerol, and disrupted by sonication on ice. The cell debris was removed by centrifugation and the supernatant was loaded onto a HisTrap FF column and eluted with a linear gradient of 50∼500 mM imidazole. The collected protein fractions were examined by electrophoresis in a 12% sodium dodecylsulphate-polyacrylamide gel electrophoresis (SDS-PAGE). The purified Sis-HhH-GPD protein was dialyzed against storage buffer (20 mM Tris-HCl, pH 8.0, 50 mM NaCl, 1 mM DTT and 50% glycerol), and stored at −80°C. The Sis-HhH-GPD protein concentration was calculated by measuring an absorbance at 280 nm with a theoretical molar extinction coefficient of 55 350 M^−1^ cm^−1^.

### Predicting model structure

We predicted a model structure of Sis-HhH-GPD using AlphaFold3 (39). The predicted structure was then superimposed onto the structure of human 8-oxoguanine DNA glycosylase (PDB code 1HU0) in a complex with double-stranded DNA (dsDNA) (40) via the HhH-GPD region, incorporating the dsDNA coordinates into the predicted structure. 1-meA base was manually constructed by Coot (41). The Fe-S cluster molecule was subsequently added to the predicted structure according to the homologous structure of *E. coli* EndoIII (PDB: 2ABK) (24). Final quality of the predicted structure was validated (Table S2). A structural figure was prepared by PyMOL (https://www.pymol.org/).

### Construction, expression and purification of the sis-HhH-GPD mutant proteins

Eleven Sis-HhH-GPD mutants (W57A, E134A, S152A, Y154A, R157A, R161A, R200A, C203A, C210A, C213A and C219A) were engineered by using a Site-directed Mutagenesis Kit (Transgen, China) starting from the wild-type (WT) plasmid harboring the *SiRe_0278* gene. Sequences of mutagenic primers are summarized in Table S1. PCR reactions were set up following the manufacturer's instructions. Each desired mutation was verified by sequencing. The Sis-HhH-GPD mutant proteins were expressed, purified and quantified as described for the WT protein.

### Circular dichroism measurements

The WT and mutant Sis-HhH-GPD proteins were dialyzed against 50 mM phosphate-buffered saline (PBS) buffer (pH 7.5) for circular dichroism (CD) analysis. The protein (0.15 mg/ml) was added to a cuvette of path length 0.2 cm for CD scanning. Using a J-810 spectropolarimeter, the spectra were collected at a scanning rate of 50 nm/min at 25°C and recorded from 200 to 250 nm wavelength. The CD spectral data were calculated as the mean residue ellipticity [θ]. Using Origin software, the CD wavelength spectra were generated. The triplicate spectrum readings were collected for each sample.

### DNA cleavage assays

Unless stated otherwise, DNA cleavage assays were performed in a reaction (10 μl) containing 20 mM Tris–HCl, pH 8.0, 1 mM DTT, 8% glycerol, 100 nM 5′-Hex-labeled single-stranded DNA (ssDNA) with 1-meA and 1000 nM Sis-HhH-GPD at 70°C for 30 min. The reactions were stopped by the addition of 10 μl of stop solution containing 98% formamide and 20 mM ethylenediaminetetraacetic acid (EDTA) to the reaction mixture. After electrophoresis in a denaturing 15% polyacrylamide gel containing 8 M urea in 0.5 × Tris-Borate-EDTA buffer, the cleaved product was visualized with a molecular image analyzer (PharosFx System, BioRad) and the quantitative analysis was performed with ImageQuant software. All DNA glycosylase assays were performed in triplicate. Statistical analysis of significant difference at the 5% level was performed by SPSS software (IBM SPSS Statistics version 19.0).

Alkylated DNA was prepared via growing the *E. coli* DH5*a* cells in LB medium in the presence of MMS. *E. coli* DH5*a* strain was inoculated into LB medium containing 5.0 mM MMS. After overnight (∼18 h), the cells were harvested by centrifuge (12 000 rpm, 3 min) at room temperature and then the genomic DNA was extracted from the collected cells using the Mini-Genomic DNA Extraction Kit (TIANGEN, China). Alkylated DNA created by MMS was used as substrate to determine the Sis-HhH-GPD activity. Using 150 ng alkylated DNA as substrate, DNA cleavage reactions were performed and stopped as described above. The genomic DNA extracted from the collected *E. coli* DH5*a* cells without MMS was used as a control. The cleaved product was separated with 1% agarose gel electrophoresis.

### Biochemical characterization assays

We used 1-meA-containing ssDNA as substrate to investigate optimal temperature, optimal pH, divalent ion and salt requirements of the Sis-HhH-GPD activity and its thermostability. The optimal temperature of the enzyme activity was determined by performing DNA cleavage reactions at 30°C, 40°C, 50°C, 60°C, 70°C, 80°C and 90°C for 30 min, respectively. Its thermostability was evaluated by heating the enzyme protein at 80°C, 85°C, 90°C and 95°C for 20 min prior to the glycosylase assays. The optimal pH for the Sis-HhH-GPD activity was investigated by performing the reactions at various pHs ranging from 6.0 to 11.0 using different buffers at 20 mM final concentration: sodium phosphate-NaOH buffer for pH 6.0 and pH 7.0; Tris–HCl buffer for pH 8.0; and Gly-NaOH buffer pH 9.0, pH 10.0 and pH 11.0. The divalent ion requirement of the Sis-HhH-GPD activity was estimated by performing DNA cleavage reactions with 1 mM of Mg^2+^, Mn^2+^, Ca^2+^, Zn^2+^, Co^2+^, Ni^2+^ or Cu^2+^. Last, the optimal salinity for the Sis-HhH-GPD activity was determined by performing DNA cleavage reactions at varied NaCl concentrations ranging from 50 to 1000 mM. All assays were done in triplicate.

### Construction of *ΔSiRe_0278* strain

As described previously, we knocked out the *SiRe_0278* gene using a pMID strategy (42). The sequences of the left-arm, right-arm and spacer primers used for constructing *ΔSiRe_0278* strain were listed in Table S3. As described previously, *S. islandicus* REY15A (E233S) was employed as a host and cultured (42). We used uracil-free MTSV medium [mineral salts (M), 0.2% (*w/v*) tryptone (T), 0.2% (*w/v*) sucrose (S) and a mixed vitamin solution (V)] solidified with 0.8% phytagel to select the transformants after electro-transformation (42). Using counter-selection, the knockout colonies were screened by spreading the cells onto a plate of MTSV medium supplemented with 5-FOA (50 μg/ml) and uracil (10 μg/ml) (43). *ΔSiRe_0278* strain was verified by PCR using the flanking and gene primers.

### MMS sensitivity assay and growth curve of *ΔSiRe_0278* and WT strains

*ΔSiRe_0278* and WT strains were cultured in MTSV medium at 75°C until OD_600_ value reached 0.4. The culture was diluted with 10^1^-, 10^2^-fold, 10^3^-fold and 10^4^-fold and then 7.5 μl of the diluted cultures was added to on MTSV solid medium containing a final MMS concentration of 0.25, 0.5 and 1.00 mM, respectively. The MMS plates were placed at a 75°C incubator for 7 days. On the other hand, the fresh cultures of *ΔSiRe_0278* and WT strains were inoculated into MTSV medium with or without a final concentration of 1.00 mM MMS. And then the culture continued to be shaken at 75°C for 108 h. The OD_600_ values were recorded to generate a growth curve. Statistical analysis was performed as described above.

### Genetic complementary analysis

The WT and mutant *SiRe_0278* genes in the pET-28at_Plus vectors encoding the WT and mutant Sis-HhH-GPD proteins (C203A, C210A, C213A and C219A) were sub-cloned into the vector pSeSD by using the forward and reverse primers (Table S3) as described above. The constructed pSeSD-SiRe_0278 vectors were transformed into *ΔSiRe_0278* strain by electro-transformation as described above. The transformants were verified with amplifying the *SiRe_0278* gene by PCR. The cells harboring the WT and mutant pSeSD-*SiRe_0278* vectors were grown in MTSV at 75°C for 36 h. When the OD_600_ nm values reached 0.2, 2.0 mM MMS was added to the cultures. No MMS was added as a control. Growth curves were generated as described above. Besides, 0.2% (*w/v*) sucrose/arabinose was added to induce the *SiRe_0278* gene expression when the OD_600_ nm values reached 0.2. Statistical analysis was performed as described above. SDS-PAGE was performed to verify whether or not the WT and mutant *SiRe_0278* genes in the vector pSeSD were expressed.

## Results

### The genome of *S. islandicus* REY15A encodes a novel HhH-GPD family protein

**Figure 1.**
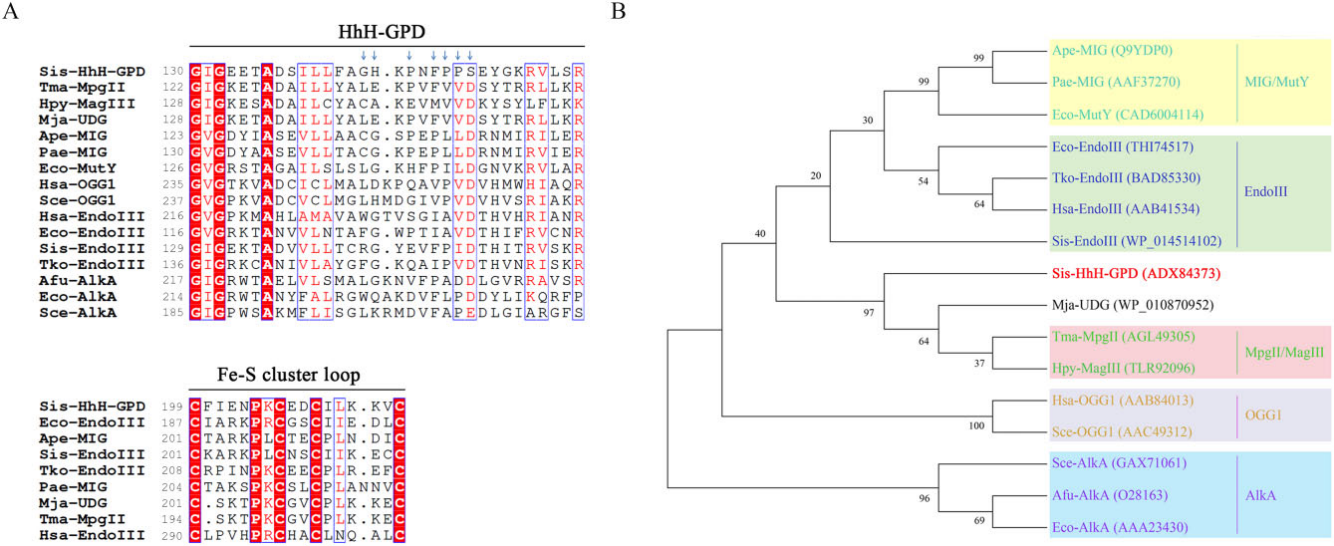
Sequence alignment and phylogenetic analysis of Sis-HhH-GPD. (**A**) Sequence alignment of an HhH-GPD motif and Fe-S cluster loop of Sis-HhH-GPD with other HhH-GPD homologs. Sis-HhH-GPD: *S. islandicus* (ADX84373); Tma-MpgII: *T. maritima* (AGL49305); Hpy-MagIII: *H. pylori* (TLR92096); Mja-UDG: *M. jannaschii* (WP_010870952); Ape-MIG: *Aeropyrum pernix* (sp|Q9YDP0.2); Pae-MIG: *Pyrobaculum aerophilum* (AAF37270); Eco-MutY: *E. coli* (CAD6004114); Hsa-OGG1: *Homo sapiens* (AAB84013); Sce-OGG1: *Saccharomyces cerevisiae* (AAC49312); Eco-EndoIII: *E. coli* (THI74517); Sis-EndoIII: *S. islandicus* (WP_014 514 102); Tko-EndoIII: *Thermococcus kodakarensis* (BAD85330); Hsa-EndoIII: *H. sapiens* (AAB41534); Afu-AlkA: *A. fulgidus* (sp|O28163); Eco-AlkA: *E. coli* (AAA23430); Sce-AlkA: *S. cerevisiae* (GAX71061). Similar residues are boxed and same residues are shaded in red. (**B**) A phylogenetic tree of Sis-HhH-GPD and other HhH-GPD homologs. A total of above 26 HhH-GPD superfamily proteins were used to construct a phylogenetic tree by MEGA X software.

A putative HhH-GPD family protein from *S. islandicus* REY15A (Genbank accession number: ADX84373) encoded by the *SiRe_0278* gene possesses a conserved HhH-GPD signature motif of HhH-GPD family proteins (Figure 1A), which includes the members from HP0602 superfamily, PRK13913 superfamily and HP0602 superfamily (Supplementary Figure S1). Besides, Sis-HhH-GPD has a Fe-S cluster loop that is coordinated with four conserved cysteine residues at its C-terminus, which is present in partial members of HhH-GPD family proteins (Figure 1A). Interestingly, Sis-HhH-GPD possesses a serine residue at the position of the invariable aspartate/glutamine in the conserved GPD loop of the HhH-GPD family proteins. Besides, Sis-HhH-GPD has a glutamine residue (E134) in HhH-GPD motif as observed for *Thermotoga maritima* MpgII, *Helicobacter pylori* 3-methyladenine DNA glycosylase III (Hpy-MagIII) and *Methanocaldococcus jannaschii* uracil DNA glycosylase (Mja-UDG), but the corresponding residue in EndoIII, OGG1, AlkA, MIG (mismatch glycosylase) and MutY is Lys, Lys, Trp, Tyr and Ser (Figure 1A), respectively. Thus, Sis-HhH-GPD might be a novel DNA glycolsyase with biological function distinct from other HhH-GPD homologs.

To probe its evolutionary relation with other HhH-GPD family proteins, we constructed a phylogenetic tree using sequences of Sis-HhH-GPD and other 25 HhH-GPD homologs from different organisms. As shown in Figure 1B, we found that Sis-HhH-GPD is clustered into a separate branch, which is close to Mja-UDG, Tma-MpgII and Hpy-MagIII, but far from other HhH-GPD proteins, such as OGG1, AlkA, MIG and EndoIII. Taken together, we proposed that Sis-HhH-GPD is a novel HhH-GPD protein.

To clarify biochemical function of the protein, we cloned the gene encoding the Sis-HhH-GPD protein into an expression vector pET28a_Plus and then a recombinant plasmid was transformed into *E. coli* BL21(DE3) pLysS cells. The Sis-HhH-GPD protein was successfully induced for expression with the addition of IPTG (Supplementary Figure S2). Using Ni column purification, the recombinant Sis-HhH-GPD protein was successfully purified to be homogeneity as a ∼26 kDa with a His-tagged protein at its N-terminus (Supplementary Figure S2).

### Removal of 1-meA from DNA by Sis-HhH-GPD

To confirm what damaged base in DNA Sis-HhH-GPD can act on, we employed U-, I-, 8-oxoG, Tg-, 1-meA-containing ssDNA and normal ssDNA as substrate to perform its DNA cleavage reactions at 70°C. DNA cleavage products were further treated with or without the hot 0.5 M NaOH (95°C) to verify whether Sis-HhH-GPD is a mono-functional or bi-functional DNA glycosylase.

**Figure 2.**
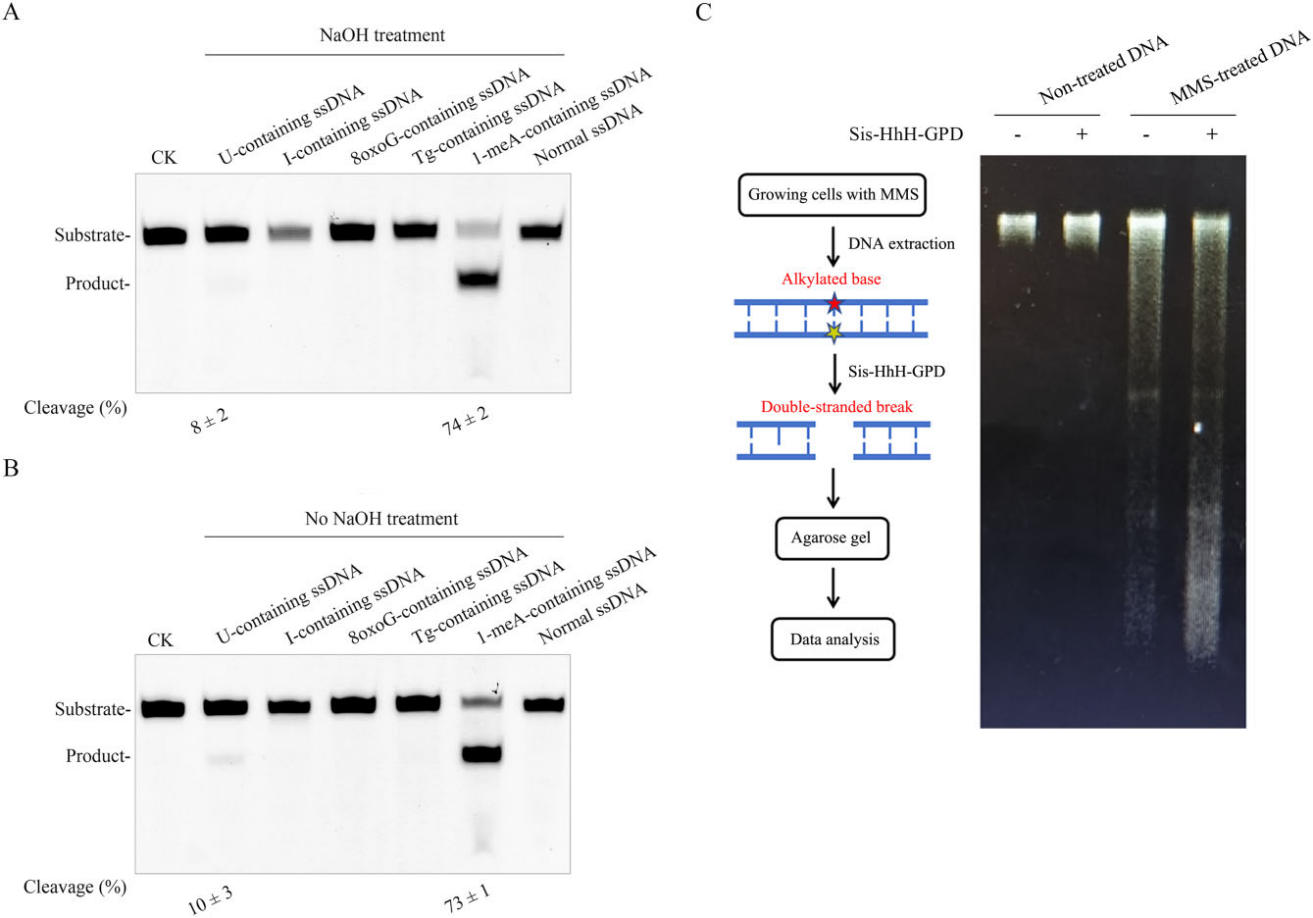
Sis-HhH-GPD can excise alkylated base from DNA. Various DNA substrates containing U, I, 8oxoG, Tg or 1-meA were used to perform DNA cleavage reactions in the presence of Sis-HhH-GPD at 70°C for 30 min. (**A**) The cleaved product was treated with the hot 0.5 M NaOH (95°C 5 min). CK: the reaction without Sis-HhH-GPD. (**B**) The cleaved product was treated without the hot 0.5 M NaOH (95°C 5 min). CK: the reaction without Sis-HhH-GPD. (**C**) Sis-HhH-GPD can excise alkylated bases from DNA created by 5.0 mM MMS; 5.0 mM MMS was added to the LB medium for culturing the *E. coli* cells. Total genomic DNA was extracted from the MMS-treated cells. Both alkylated pair bases in DNA were cleaved by Sis-HhH-GPD to form double-stranded breaks (DSBs). DNA fragments containing DSBs were separated by agarose gel electrophoresis. Genomic DNA extracted from the *E. coli* cells without MMS was used as control.

As shown in Figure 2A and B, the ssDNA substrate harboring I, 8oxoG or Tg remained intact after incubating them with Sis-HhH-GPD no matter what the reaction product was treated with the hot NaOH or not. Furthermore, only a tiny amount of the cleaved product was observed when using U-containing ssDNA as substrate. By comparison, we observed that 74% 1-meA-containing ssDNA substrate was cleaved by Sis-HhH-GPD at 70°C with the hot NaOH treatment (Figure 2A). Likewise, similar cleavage (73%) was observed for the reaction without the hot NaOH treatment (Figure 2B), thereby demonstrating that Sis-HhH-GPD is a bi-functional DNA glycosylase that can remove 1-meA from DNA at high temperature.

Next, we examined the effect of enzyme concentration on the Sis-HhH-GPD activity. As shown in Supplementary Figure S3A, the cleavage efficiency increased as the enzyme concentrations increased. At 1000 nM, maximum cleavage efficiency reached about 70%. Even further increase in enzyme concentration did not still increase the cleavage efficiency (data not shown), suggesting that 1-meA-containing ssDNA is not be a preferred substrate for Sis-HhH-GPD. However, this enzyme cannot act on normal ssDNA (Supplementary Figure S3B). On the other hand, Sis-HhH-GPD can cleave 1-meA-contraining dsDNA (1-meA:T) with the slightly reduced efficiency relative to 1-meA-containing ssDNA cleavage (Supplementary Figure S3C). Likewise, no cleavage was detected when using normal dsDNA as substrate (Supplementary Figure S3D). Thus, Sis-HhH-GPD is designated to be an alkylated DNA glycosylase that can excise 1-meA from DNA.

We also tested whether Sis-HhH-GPD can cleave 1-meA:C, 1-meA:G and 1-meA:A-containing dsDNA. As shown in Supplementary Figure S4, we observed similar cleavage efficiencies regardless of which base paired with 1-meA. Thus, these observations might be congruent with removing an alkylated base by flipping it out from DNA and then cleaving it by Sis-HhH-GPD.

### Sis-HhH-GPD can excise alkylated base from DNA created by MMS

To investigate whether Sis-HhH-GPD excises other alkylated bases from DNA in addition to 1-meA, we prepared alkylated DNA by extracting the genomic DNA from the cultured *E. coli* DH5*a* cells in the presence of 5.0 mM MMS and used it as substrate to perform DNA cleavage reaction of Sis-HhH-GPD (Figure 2C). In the control reaction where using the genomic DNA prepared with the cultured *E. coli* DH5*a* cells in the absence of 5.0 mM MMS as substrate, this substrate remained intact after incubating it with Sis-HhH-GPD (Figure 2C), demonstrating that this enzyme is inactive to normal genomic DNA. When using the DNA prepared from the MMS-treated *E. coli* DH5*a* cells as substrate, small smear DNA fragments were produced after incubating at 70°C for 30 min. This arises from a possibility that alkylated bases in DNA created by MMS have not been completely repaired in *E. coli*, thus leading to single-stranded breaks (SSBs). And then further heating at 70°C converts the resulting SSBs to DSBs. Compared with the control reaction, more small smear DNA fragments were generated after incubating Sis-HhH-GPD with DNA substrate (Figure 2C), suggesting that this protein can remove alkylated bases from DNA created by MMS.

### Biochemical characteristics of sis-HhH-GPD

**Figure 3.**
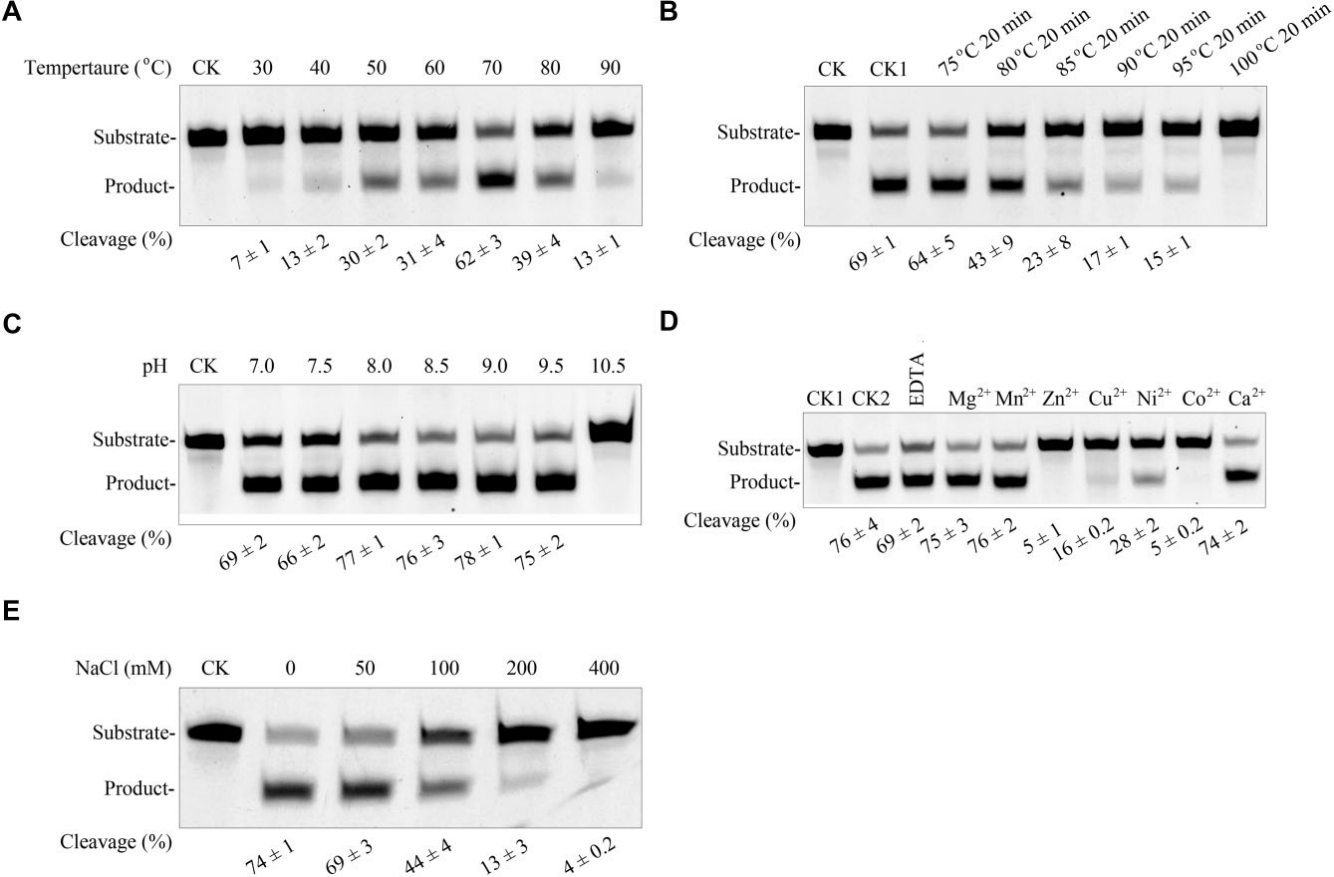
Biochemical characteristics of cleaving 1-meA-containing ssDNA by Sis-HhH-GPD. DNA cleavage reactions were performed by Sis-HhH-GPD using 1-meA-containing ssDNA as substrate under various conditions. (**A**) The optimal temperature assay results. (**B**) The thermo-tolerance assay results. (**C**) The optimal pH assay results. (**D**) The effects of various divalent metal cations. (**E**) The effect of NaCl. CK in panels (A), (B), (C) and (E), and CK1 in panel (D): the reaction without Sis-HhH-GPD; CK1 in panel (B): the reaction with non-heated Sis-HhH-GPD; CK2 in panel (D): the reaction without a divalent cation.

Since Sis-HhH-GPD can excise 1-meA from ssDNA, this substrate was used in all further experiments to probe its biochemical characteristics. Our data demonstrate that the optimal temperature for the Sis-HhH-GPD activity is 70°C (Figure 3A), which is close to the optimal growth temperature of the protein host. Additionally, Sis-HhH-GPD retained about 15% cleavage after incubating 20 min at 90°C or 95°C, and showed no activity when heated 20 min at 100°C, displaying limited thermostability (Figure 3B). Furthermore, Sis-HhH-GPD can remove 1-meA from ssDNA at various pHs ranging from 6.0 to 9.5 (Figure 3C). Maximum cleavage efficiencies were observed in a pH range from 8.0 to 9.5 (Supplementary Figure S5), suggesting that the optimal pH of the Sis-HhH-GPD activity is 8.0–9.5.

The control reaction with 10 mM EDTA (Figure 3D) showed that Sis-HhH-GPD retained the cleavage efficiency similar to the control reaction without EDTA (69% versus 76% respectively), suggesting that a divalent metal ion is not required for the enzyme activity. Mg^2+^, Mn^2+^ and Ca^2+^ marginally increased cleavage efficiencies of Sis-HhH-GPD (Figure 3D). In contrast, DNA cleavage by Sis-HhH-GPD was inhibited with Zn^2+^, Cu^2+^, Co^2+^ and Ni^2+^ (Figure 3D).

Last, the Sis-HhH-GPD activity was gradually inhibited with increasing NaCl concentrations (Figure 3E). In the presence of 200 mM NaCl, the enzyme retained only 13% cleavage. Almost no cleavage was detected at 400 mM NaCl. Therefore, these results demonstrate that NaCl is not required for Sis-HhH-GPD to excise 1-meA from DNA and high concentration NaCl inhibits its activity.

### Sis-HhH-GPD has AP lyase activity

As mentioned above, our data show that Sis-HhH-GPD is a bi-functional DNA glycosylase. Thus, we further investigated whether Sis-HhH GPD has AP lyase activity. AP-containing ssDNA was prepared by using a mono-functional *E. coli* UDG to remove U-containing ssDNA. As shown in Supplementary Figure S6, 24% cleavage can already be observed without Sis-HhH-GPD, which arises from the fact that the reaction temperature of 70°C leads to a partial disruption of the AP sites. Interestingly, the addition of Sis-HhH-GPD to the AP-containing ssDNA generated by *E. coli* UDG increased the cleavage percentage (Supplementary Figure S6), thereby demonstrating that the protein can cleave a phsopshodietster of AP-containing ssDNA.

### A predicted structure of Sis-HhH-GPD

**Figure 4.**
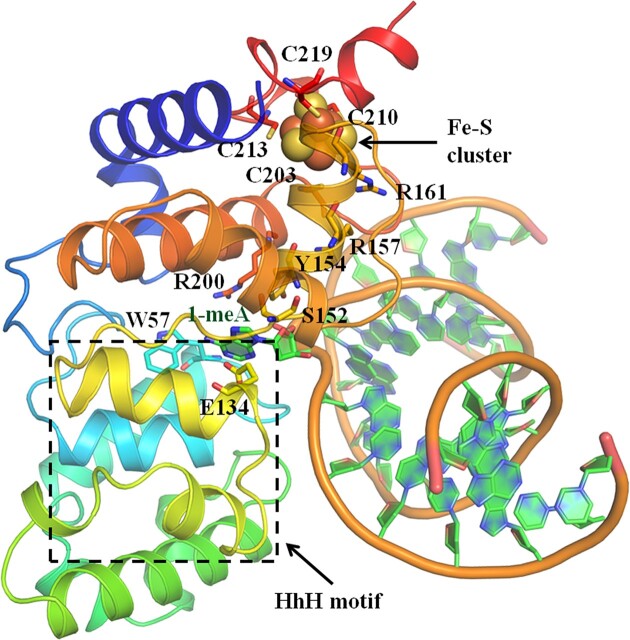
A predicted structure of Sis-HhH-GPD protein. A model structure of Sis-HhH-GPD was predicted by AphlaFold3. 1-meA-containing dsDNA and Fe-S cluster were added to the predicted structure to form the complex structure. The potential residues in the complex structure used for 1-meA removal are labeled with sticks. The details are described in text.

Using AlphaFold3, we predicted a structure of Sis-HhH-GPD and added dsDNA containing 1-meA to the predicted structure by Pymol (Figure 4). As already seen in the protein sequence alignment (Figure 1A), S152 is distinct from a corresponding Asp residue in other HhH-GPD proteins and close to 1-meA base in the predicted structure of Sis-HhH-GPD. Besides, E134 is conserved in Mja-UDG, Tma-MpgII and Hpy-MagIII but not in other HhH-GPD family proteins and also next to 1-meA. On the other hand, R157 and R161 in the GPD motif of Sis-HhH-GPD are near to the DNA substrate, suggesting that they might bind DNA. As shown in the structure (Figure 4), four cysteine residues (C203A, C210A, C213A and C219A) associate with a Fe-S cluster loop. Additionally, we observed that W57, Y154 and R200 surround the 1-meA base, suggesting that these residues might be involved in1-meA removal.

To uncover functional roles of these eleven residues of Sis-HhH-GPD in 1-meA removal, we mutated them to be alanine. All Sis-HhH-GPD mutant proteins were expressed and purified to be homogeneity as described for the WT protein (Supplementary Figure S7).

### Secondary structures of the WT and mutant sis-HhH-GPD

**Figure 5.**
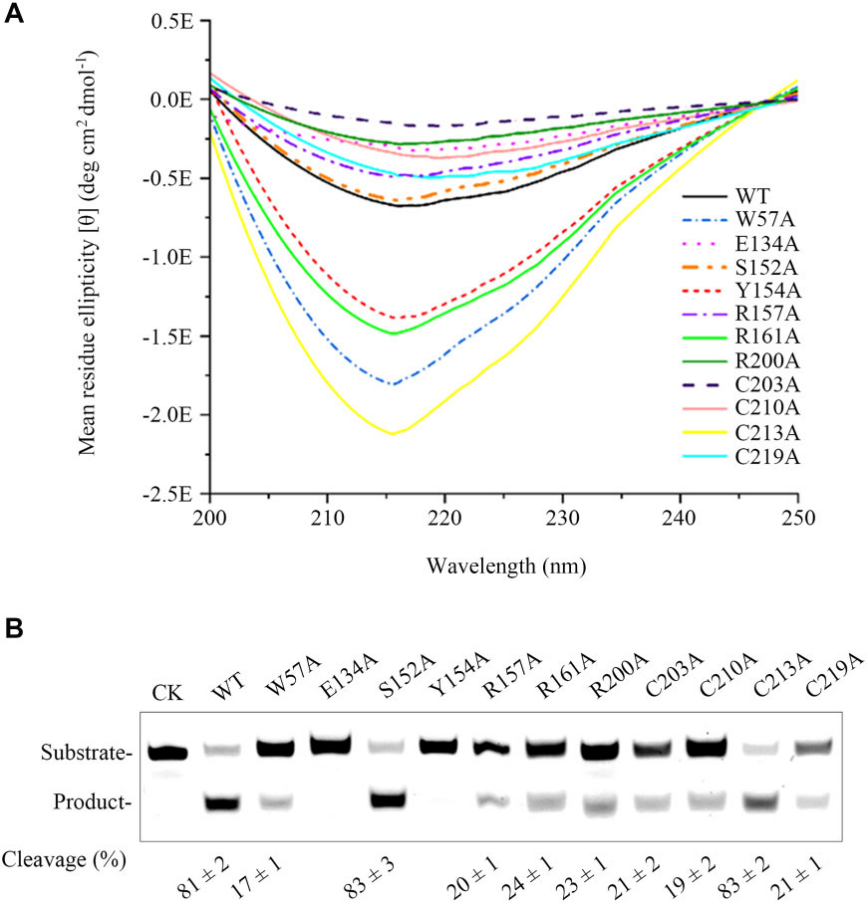
Comparison of secondary structures and DNA cleavage of the WT and mutant Sis-HhH-GPD proteins. (**A**) Secondary structures of the WT and mutant Sis-HhH-GPD proteins by using CD analyses. Secondary structures of the WT and mutant Sis-HhH-GPD proteins are labeled with different colored lines. (**B**) DNA cleavage of the WT and mutant Sis-HhH-GPD. Using 100 nM 1-meA-containing ssDNA, DNA cleavage reactions of the WT and mutant Sis-HhH-GPD were performed at 1000 nM at 70°C for 30 min. CK: the reaction without Sis-HhH-GPD.

To investigate whether these eleven substitutions disrupt the overall secondary structure, we conducted the CD assays of the WT and mutant Sis-HhH-GPD proteins. As shown in Figure 5A, the S152A mutant exhibited the almost same structure as the WT protein, demonstrating that the replacement of S152 with Ala does not affect the overall structure of the enzyme. Compared with other mutants, the C213A mutant displayed maximum difference of secondary structure relative to the WT protein, indicating that the mutation of C213 to Ala disrupts the overall structure of the enzyme to the greatest extent. Furthermore, the W57A, R161A, Y154A, C203A, R200A, E134A, C210A, C219A and R157A substitutions changed the overall secondary structure of the enzyme with varied degrees.

### DNA cleavage activities of the WT and mutant sis-HhH-GPD

Cleavage efficiencies of these eleven constructed Sis-HhH-GPD mutants were investigated by performing DNA cleavage reactions using 1-meA-containing ssDNA as substrate at 70°C. Compared with the WT protein, the E134A and Y154A mutants had no cleavage activity (Figure 5B), demonstrating that the mutations of E134 and Y153 to alanine lead to complete loss of the enzyme activity. By comparison, the S152A and C213A mutants displayed the WT protein cleavage, indicating that the substitutions of S152 and C213 with alanine have marginal effect on the enzyme activity. All other mutants, e.g. W57A, R157A, R161A, R200A, C203A, C210A and C219A mutants, displayed the reduced cleavage efficiencies with varied degrees, suggesting that the replacements of W57, R157, R161, R200, C203, C210 and C219 with alanine partially affect the enzyme activity. Therefore, residues E134 and Y154 are essential for 1-meA removal by Sis-HhH-GPD, and a combination of W57, R157, R161, R200, C203, C210 and C219 plays a role in assisting in catalysis.

### Elevated MMS sensitivity of *ΔSiRe_0278* strain

**Figure 6.**
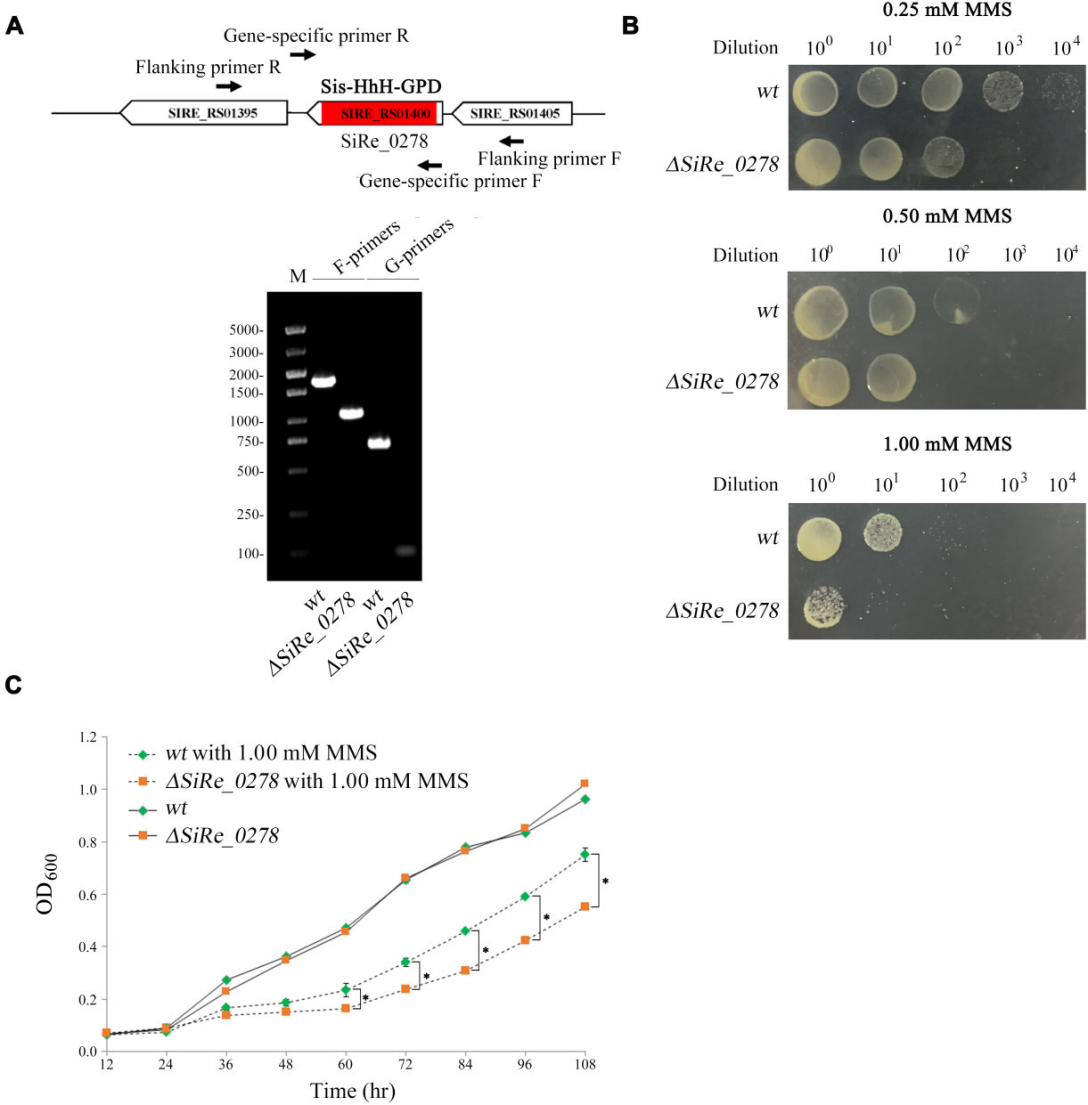
Sis-HhH-GPD is responsible for repair of alkylated DNA created by MMS in the *S. islandicus* REY15A cells. (**A**) Construction of *ΔSiRe_0278* strain. (**B**) Sensitivity assays of *ΔSiRe_0278* and WT strains to MMS. (**C**) Growth curves of *ΔSiRe_0278* and WT strains in the absence and presence of 1.0 mM MMS. ‘*’ indicated *P* < 0.05.

To reveal physiological function of Sis-HhH-GPD *in vivo*, we used homologous recombination (HR) method via uracil selection and 5-FOA counter-selection to construct ***Δ****SiRe_0278* strain (42). The constructed ***Δ****SiRe_0278* strain was verified by PCR analysis using the flanking primers since a size of the amplified DNA band using the genomic DNA of ***Δ****SiRe_0278* strain was smaller than that of using the genomic DNA of WT strain (Figure 6A). Using the gene-specific primers, no corresponding band was amplified with the genomic DNA of ***Δ****SiRe_0278* strain by PCR (Figure 6A), but one corresponding band appeared with the genomic DNA of WT strain, thereby demonstrating that ***Δ****SiRe_0278* strain was successfully constructed.

After obtaining ***Δ****SiRe_0278* strain, we investigated its sensitivity to MMS using WT strain as control. As MMS concentrations increased, both WT and *ΔSiRe_0278* stains showed sensitivity to MMS (Figure 6B). Notably, *ΔSiRe_0278* stain displayed the stronger sensitivity to MMS than WT strain in the presence of MMS with all the tested concentrations (Figure 6B), thus suggesting that Sis-HhH-GPD encoded by the *SiRe_0278* gene is responsible for alkylated DNA repair created by MMS.

### Slowed growth of *ΔSiRe_0278* strain in the presence of MMS

Additionally, we also tested the growth of WT and *ΔSiRe_0278* strains with or without MMS. In the absence of MMS, no distinct growth was observed between WT strain and *ΔSiRe_0278* strain, showing that *SiRe_0278* is non-essential for cell viability (Figure 6C). By comparison, *ΔSiRe_0278* strain displayed the significantly slower growth than WT strain when the growth time was equal to or longer than 16 h in the presence of 1.0 mM MMS (Figure 6C), thus further confirming an essential role of Sis-HhH-GPD in repair alkylated bases in DNA created by MMS.

### Physiological roles of sis-HhH-GPD revealed by genetic complementary analyses

**Figure 7.**
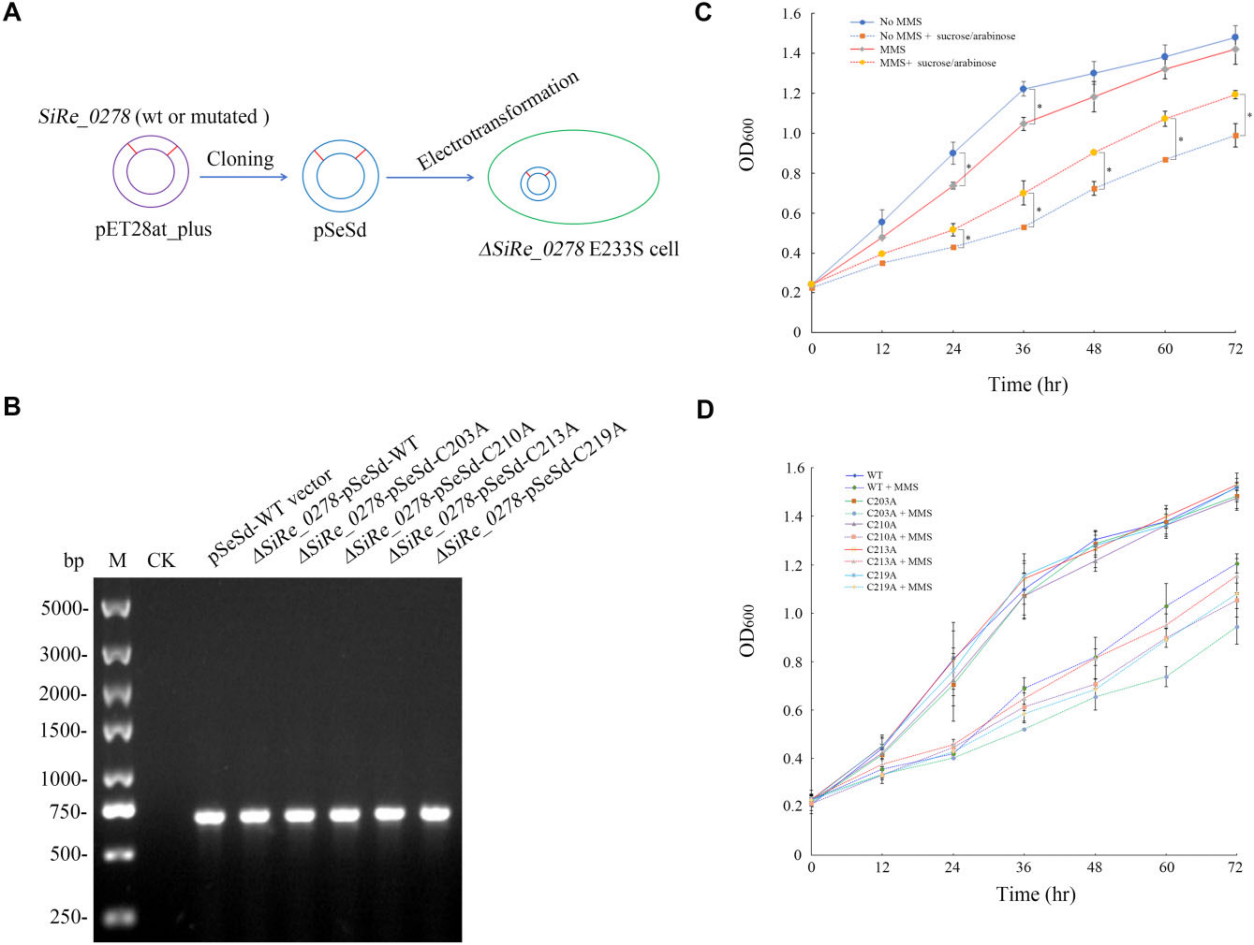
Genetic complementary analyses of the WT and mutant Sis-HhH-GPD proteins. (**A**) Schematic diagram for constructing the recombinant *ΔSiRe_0278* strains harboring the WT and mutant pSeSd-*SiRe_0278* plasmids. (**B**) PCR verification of the WT and mutant recombinant *ΔSiRe_0278* strains. (**C**) Growth curves of the recombinant *ΔSiRe_0278* strains with or without the addition of 0.2% (*w/v*) sucrose/arabinose in the presence or absence of 2.0 mM MMS. (**D**) Growth comparison of the WT and mutant recombinant *ΔSiRe_0278* strains with the addition of 0.2% (*w/v*) sucrose and arabinose in the presence of 2.0 mM MMS.

The expression vector pSeSd can be used for protein expression in the *S. islandicus* REY15A cells (36,37). To uncover further physiological role of Sis-HhH-GPD, we cloned the *SiRe_0278* gene into the vector pSeSd and then transformed the recombinant plasmid into the *ΔSiRe_0278* cells (Figure 7A). The constructed *ΔSiRe_0278* cells harboring the recombinant pSeSd plasmid was verified by amplifying the *SiRe_0278* gene by PCR (Figure 7B). As shown in Figure 7C, the *ΔSiRe_0278-*pSeSd-WT strain displayed similar growth no matter what sucrose/arabinose was added in the absence of 2.0 mM MMS except at 24 and 36 h. However, the *ΔSiRe_0278-*pSeSd-WT strain slowed the growth at 24 and 36 h in the presence of sucrose/arabinose, which arises from a possibility that the *ΔSiRe_0278* gene was expressed in the *ΔSiRe_0278* cells in the absence of MMS (Supplementary Figure S8). Compared with the addition of sucrose/arabinose, the *ΔSiRe_0278-*pSeSd-WT strain exhibited the slowed growth without the addition of sucrose/arabinose in the presence of 2.0 mM MMS at 24∼72 h, suggesting that Sis-HhH-GPD can play an essentially complementary role in repairing alkylated DNA created by MMS in the *ΔSiRe_0278* cells. Likewise, the *SiRe_0278* gene was expressed in the *ΔSiRe_0278* cells with the addition of sucrose/arabinose in the presence of 2.0 mM MMS (Supplementary Figure S8). Interestingly, the increased expression of the *SiRe_0278* gene was observed in the *ΔSiRe_0278* cells with 2.0 mM MMS relative to the expression without 2.0 mM MMS (Supplementary Figure S8), thus further confirming physiological roles of Sis-HhH-GPD.

### Physiological roles of four conserved cysteine residues in sis-HhH-GPD

As described for constructing the *ΔSiRe_0278-*pSeSd-WT strain, we also constructed the *ΔSiRe_0278-*pSeSd-C203A, *ΔSiRe_0278-*pSeSd-C210A, *ΔSiRe_0278-*pSeSd-C213A and *ΔSiRe_0278-*pSeSd-C219A strains. As shown in Figure 7D, the *ΔSiRe_0278-*pSeSd-C213A strain displayed similar growth to the *ΔSiRe_0278-*pSeSd-WT strain with the addition of 0.2% (*w/v*) sucrose/arabinose in the presence of 2.0 mM MMS, which is consistent with the observation that the C213A mutant has the WT protein activity. By contrast, the *ΔSiRe_0278-*pSeSd-C203A, *ΔSiRe_0278-*pSeSd-C210A and *ΔSiRe_0278-*pSeSd-C219A strains exhibited the significantly slowed growth at 48, 60 and 72 h relative to the *ΔSiRe_0278-*pSeSd-WT strain (Supplementary Figure S9), which is also consistent with the observations that the C203A, C210A and C219A mutants have the reduced activities relative to the WT protein. Like the WT *SiRe_0278* gene, the mutated *SiRe_0278* genes encoding the C203A, C210A, C213A and C219A mutants were expressed in the *ΔSiRe_0278* cells in the presence of 2.0 mM MMS (Supplementary Figure S10). On the other hand, no growth difference was observed between the *ΔSiRe_0278-*pSeSd-WT strain and these four mutant strains with the addition of 0.2% (*w/v*) sucrose/arabinose in the absence of 2.0 mM MMS. Thus, functional roles of these four conserved cysteine residues that associate with the Fe-S cluster in Sis-HhH-GPD *in vitro* is accordance with their physiological roles *in vivo*.

## Discussion

### Sis-HhH-GPD is a bi-functional alkylated DNA glycosylase

Since alkylated bases in DNA are mutagenic and/or toxic, cells have evolved several pathways for repair of these damaged bases to restore original genetic information. To date, our understanding on how Archaea, especially Crenarchaea, repair alkylated bases in DNA has not fully been clarified. 1-meA is generated upon exposure to alkylating agents (44–46) and has been shown to be highly cytotoxic since it can block DNA replication and transcription (47). It has been confirmed that AlkB proteins in *E. coli* and mammals are responsible for repair of 1-meA in DNA (48,13,49). However, no AlkB protein is encoded in genomic DNA of hyperthermophilic Crenarchaea, suggesting that 1-meA would be removed from DNA via distinct pathways after exposed to DNA damaging agents.

In this work, we demonstrate that Sis-HhH-GPD can remove 1-meA from DNA and alkylated bases from DNA created with MMS, thereby verifying that this protein is an alkylated DNA glycosylase. Since Sis-HhH-GPD displays only about 70% activity for cleaving 1-meA-containing ssDNA and similar activity for cleaving 1-meA-containing dsDNA, we assumed that 1-meA-containing DNA is not a preferred substrate for the enzyme, and other alkylated bases of DNA, such as DNA with 3-meA or 7-meC, might be preferred. Overall, our findings on alkylated base removal from DNA by Sis-HhH-GPD provide important evidence for understanding repair mechanism of alkylated bases in DNA from the hyperthermophilic *Saccharolobus* cells.

Although they can excise 1-meA from DNA, Sis-HhH-GPD and *A. fulgidus* AlkA (Afu-AlkA) vary with sequence, evolutionary relationship and mono-/bi-functionality. Firstly, E134 and S152 of Sis-HhH-GPD are analogous to W221 and D238 of Afu-AlkA (Figure 1A), respectively. Furthermore, our constructed phylogenetic tree demonstrates that Sis-HhH-GPD displays a relatively far evolutionary relationship with Afu-AlkA (Figure 1B). Intriguingly, Sis-HhH-GPD is a bi-functional DNA glycosyalse as observed for *T. gammatolerans* AlkA (Tga-AlkA), which is a bi-functional DNA glycosylase that can excise I and 1-meA from dsDNA (35). In contrast with Sis-HhH-GPD and Tga-AlkA, Afu-AlkA is a mono-functional one. Overall, Sis-HhH-GPD is a novel bi-functional alkylated DNA glycosylase.

### Catalytic mechanism of sis-HhH-GPD

Analysis of amino acid sequences of Sis-HhH-GPD demonstrates that this protein possesses a highly conserved HhH-GPD motif present in other HhH-GPD superfamily proteins. As one of HhH-GPD superfamily proteins, EndoIII has a highly conserved lysine in the HhH-GPD motif, such as K120 in *E. coli* EndoIII. The ϵ-amino group in K120 functions as a nucleophile to generate a covalent Schiff base for catalysis (24). However, Sis-HhH-GPD lacks this catalytic lysine residue and instead harbors a residue E134, which is essential for 1-meA removal. E132 of Mja-UDG is analogous to E134 of Sis-HhH-GPD. While Mja-UDG is a mono-functional DNA glycosylase, Sis-HhH-GPD is a bi-functional one. The mutation of E132 to Lys converts a mono-functional Mja-UDG to be a bi-functional enzyme (50), suggesting that Sis-HhH-GPD and Mja-UDG vary with damaged base removal mechanisms. Besides, HhH-GPD superfamily proteins have a conserved aspartate residue, such as D138 in *E. coli* EndoIII, which is another catalytic residue (24). However, Sis-HhH-GPD still lacks the corresponding Asp residue but has a serine (S152). Mutational analyses show that S152 is not essential in Sis-HhH-GPD for catalysis. Thus, these observations suggest that Sis-HhH-GPD employs a novel mechanism to remove alkylated base from DNA distinct from EndoIII and Mja-UDG.

The crystal structure of *E. coli* AlkA shows that W272 and Y222 make contacts with the 3-meA base by stacking face-to-face (51), which potentially enhances stacking between alkylated base and their aromatic side chains. The stacking role derived from aromatic side chains of W272 and Y222 against the alkylated base provides direct assistance for base removal via the catalytic residue D238 (51). As shown in the predicted structure of the complex of Sis-HhH-GPD with DNA harboring a 1-meA (Figure 4), W57 and Y154 make contacts with 1-meA base, suggesting that they might have similar roles as observed for W272 and Y222 in *E. coli* AlkA. Mutational analyses and CD data have confirmed their assistance roles in 1-meA removal (Figure 5). Thus, we proposed that Sis-HhH-GPD utilizes the positioned planar and aromatic rings of W57 and Y154 to stack against flipping an alkylated base out and then the flipped base is cleaved by E132.

### Functional roles of residues R157, R161 and R200 of sis-HhH-GPD

The HhH-GPD motif of Sis-HhH-GPD harbors two arginines (R157 and R161), which are relatively conserved in HhH-GPD superfamily proteins, but their function remains unclear. Our mutational data show that both R157A and R161A mutants remained 20% cleavage activities (Figure 5B), suggesting that R157 and R161 are involved in catalysis. On the other hand, the predicted structure of Sis-HhH-GPD shows that R200 is close to DNA and 1-meA, and mutational data demonstrate that R200 participates in removal of 1-meA from DNA by Sis-HhH-GPD.

### Functional roles of four conserved cysteine residues of sis-HhH-GPD

Sis-HhH-GPD harbors four conserved cysteine residues that associate with a Fe-S cluster loop present in partial HhH-GPD superfamily proteins. However, whether or not this Fe-S cluster is involved in stabilization of the overall protein structure remains still unclear. Mutational studies show that C208 and C215 of *T. kodakarensis* EndoIII (Tko-EndoIII) appears to play a role in DNA cleavage in addition to the cluster formation (52). Like C208 of Tko*-*EndoIII, C205 of *Thermococcus barophilus* Ch5 EndoIII (Tba-EndoIII) is involved in DNA cleavage (53). However, C212 of Tba-EndoIII has a minor role in DNA cleavage (53), which is distinct from C215 of Tko-EndoIII. In this study, our data demonstrate that C203 of Sis-HhH-GPD, which resembles C208 of Tko*-*EndoIII and C205A of Tba-EndoIII, is involved in DNA cleavage. In contrast with C212 of Tba-EndoIII, C210 of Sis-HhH-GPD participates in DNA cleavage. However, C213 of Sis-HhH-GPD is not responsible for DNA cleavage, which is similar to C215 of Tba-EndoIII. Like C221 of Tba-EndoIII, C219 of Sis-HhH-GPD is involved in DNA cleavage. Thus, functional roles of these four conserved cysteine residues in HhH-GPD superfamily proteins vary with the protein hosts.

Besides, we used genetic complementary analyses to reveal physiological function of four conserved cysteine residues of Sis-HhH-GPD *in vivo* (Figure 7D). Interestingly, the physiological roles of four conserved cysteine residues are consistent with biochemical function *in vitro*. To our knowledge, it is the first report on physiological function of four conserved cysteine residues in HhH-GPD superfamily proteins.

### Physiological function of sis-HhH-GPD

In the present study, our genetic data demonstrated that the *SiRe_0278* gene is non-essential for cell viability, but *ΔSiRe_0278* strain displayed the higher sensitivity to MMS than WT strain (Figure 6). Besides, *ΔSiRe_0278* strain exhibited the slower growth than WT strain in the presence of MMS. Interestingly, genetic complementary analyses further confirmed physiological function of Sis-HhH-GPD *in vivo* in alkylated DNA repair (Figure 7C). Thus, we concluded that Sis-HhH-GPD is responsible for repair of DNA damage caused by MMS in the *S. islandicus* REY15A cells.

It has been shown that *Saccharolobus solfataricus* MGMT (Sso-MGMT) is a well-characterized MGMT (54–58), playing an essential role in repairing cytotoxic and mutagenic O^6^-meG via directly transferring the methyl group in the lesion to a specific cysteine in this protein as observed for other MGMTs (9,10). The *S. islandicus* REY15A cells possesses the *SiRe_0281* gene encoding a MGMT (38), which is the closest homolog of Sso-MGMT. Although Sis-MGMT has not been biochemically characterized, the verified function of Sso-MGMT *in vivo* and *in vitro* in repairing O^6^-meG in DNA allows us to propose that Sis-MGMT might be involved in O^6^-meG repair. Our genetic data support a proposal that Sis-HhH-GPD is probably involved in O^6^-meG repair. Thus, a combination of Sis-HhH-GPD and Sis-MGMT might be responsible for DNA containing O^6^-meG *in vivo*.

DpoIV from *S. solfataricus*, a typical translesion DNA polymerase belonging to the Y-family (59), can bypass O^6^-meG and 7-meG in DNA (60–62), which potentially leads to mutations. Notably, *S. islandicus* REY15A harbors the *SiRe_0236* gene, which encodes a Y-family DNA polymerase (Sis-PolY) (38). Despite not being characterized, Sis-PolY might be involved in bypassing DNA with alkylated base to synthesize DNA with an error since this PolY is the closest DpoIV homolog.

There is a possibility that both paired bases in DNA would be alkylated after DNA is exposed to alkylating agents. A DSB would occur when Sis-HhH-GPD can excise both alkylated pair bases from DNA. The resulting DSB would be repaired by HR repair. The genome of *S. islandicus* REY15A encodes the genes that are responsible for HR repair (38), among which all four genes (*mre11*, *rad50*, *herA* and *nurA*) have been confirmed to be essential for cell viability (63).

**Figure 8.**
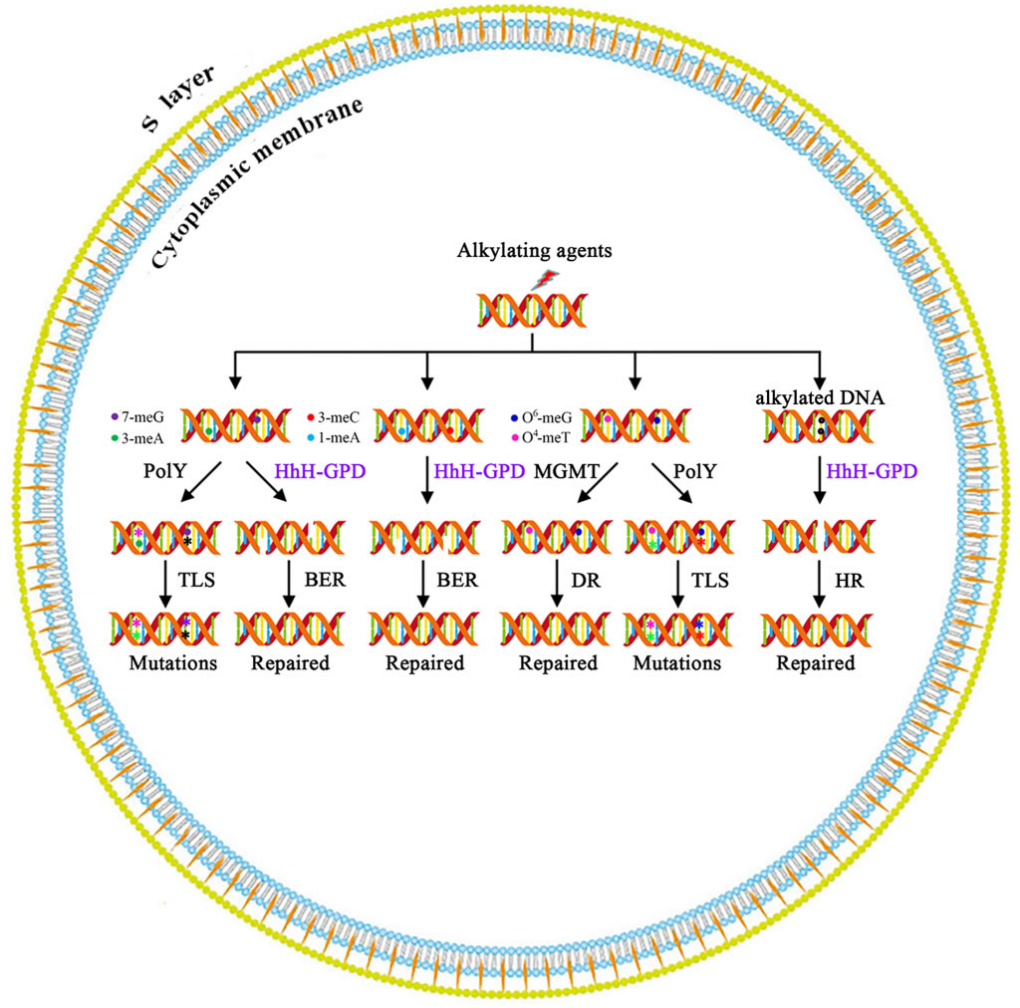
A proposed model for alkylated DNA repair in the *S. islandicus* REY15A cells. The details are described in text. BER, base excision repair; DR, direct repair; HR, homologous recombination; PolY, polymerase Y; MGMT, O^6^-methylguanine methyltransferase; TLS, translesion synthesis.

Taken together, we proposed a model for repair of alkylated bases in DNA in the *S. islandicus* REY15A cells (Figure 8). Alkylated bases in DNA can be created by endogenous and exogenous DNA alkylating agents. 1-meA, 3-meC, 7-meG and 3-meA in DNA would be recognized and excised from DNA by Sis-HhH-GPD’s glycosylase activity, thus creating an AP site. The resulting AP site by Sis-HhH-GPD could be further cleaved by its AP lyase activity, forming a modified deoxyribose residue. The following repair would be taken over by other BER enzymes or HR proteins to restore original bases in DNA strand. Additionally, Sis-PolY might be responsible for repairing DNA with 7-meG, 3-meA, O^6^-meG and O^4^-meT, which leads to potential mutations. On the other hand, Sis-MGMT can be involved in DNA with O^6^-meG or O^4^-meT.

In conclusion, we reveal for the first time that a novel HhH-GPD family protein from the hyperthermophilic crenarchaeon *S. islandicus* REY15A is a bi-functional alkylated DNA glycosylase that can remove 1-meA from DNA and alkylated bases created by MMS from DNA at high temperature. Mutational data support that E134 is essential for 1-meA removal and W57 and Y154 provide their aromatic rings to stack against the 1-meA base for base flipping-out. We also demonstrated that R157, R161 and R200 are partially involved in 1-meA removal. Besides, we observed that C203, C210 and C219 are involved in removal of 1-meA from DNA among these four cysteine residues that potentially coordinate with a Fe-S cluster loop. Notably, we first confirmed that Sis-HhH-GPD is responsible for repair of alkylated bases in DNA *in vivo*. Overall, our work provides new insight into alkylated DNA repair in *S. islandicus* REY15A by HhH-GPD family protein, which might be applicable for other hyperthermophilic Crenarchaea.

## Supplementary Material

gkaf012_Supplemental_File

## Data Availability

The data underlying this article will be shared on reasonable request to the corresponding author Dr Likui Zhang.
