# Supplementary material for: Alkylated DNA repair by a novel HhH-GPD family protein from Crenarchaea
Source: Nucleic Acids Res. 2025 Jan 22;53(2):gkaf012. doi: 10.1093/nar/gkaf012 (PMC11754123; doi:10.1093/nar/gkaf012)
Supplement: gkaf012_Supplemental_File [file gkaf012_supplemental_file.pdf]

Supplemental data

Alkylated DNA Repair by a Novel HhH-GPD Family Protein from Crenarchaea

Likui Zhang[1#], Tian Gao[1], Zheng Li[2], Cai Chen[1], Donghao Jiang[1], Youcheng Yin[1], Yaqi Zheng[1], Peng Cao[3], Yong Gong[4] and Zhihui Yang[2#]

[1]College of Environmental Science and Engineering, Yangzhou University, China

[2]College of Plant Protection, Agricultural University of Hebei, Baoding City, Hebei Province 071001, China

[3]College of Chemistry and Life Science, Beijing University of Technology, Beijing, China

[4]Beijing Synchrotron Radiation Facility, Institute of High Energy Physics, Chinese Academy of Sciences, Beijing, China

[#]Corresponding author: Dr. Likui Zhang

E-mail address: lkzhang@yzu.edu.cn

[#]Corresponding author: Dr. Yong Gong

E-mail address: yonggong@ihep.ac.cn

Corresponding author: Prof. Zhihui Yang

E-mail address: bdyzh@hebau.edu.cn

Table S1 Sequences of the oligonucleotides used in this work

Name	Sequence (5′-3′)
Sis-HhH-GPD F	CGC*GGATCC*ATGGTTCGTAAAATACTTGAC
Sis-HhH-GPD R	CCG*CTCGAG*TCACGAGGAATTTTCTCTATA
W57A F	TGGTTCAAATGTCAAGAGCGGAAATTGTAAAG
W57A R	GCTCTTGACATTTGAACCAATATTGCTGAAAT
E134A F	TGATGGTATAGGCGAAGCAACAGCTGACTCA
E134A R	GCTTCGCCTATACCATCAATGCTTAAAAGTA
S152A F	AACCAAACTTTCCACCAGCAGAGTACGGTAA
S152A R	CTGGTGGAAAGTTTGGTTTGTGACCTGCGAA
Y154A F	ACTTTCCACCATCAGAGGCCGGTAAGAGAGTA
Y154A R	GCCTCTGATGGTGGAAAGTTTGGTTTGTGACC
R157A F	CATCAGAGTACGGTAAGGCAGTATTATCTAGA
R157A R	GCCTTACCGTACTCTGATGGTGGAAAGTTTGG
R161A F	GTAAGAGAGTATTATCTGCAGTATTAGGAATT
R161A R	GCAGATAATACTCTCTTACCGTACTCTGATGG
R200A F	GAATAGTCACTGTAGGTGCAGCATTTTGTTTC
R200A R	GCACCTACAGTGACTATTCCAGCGTGTAGTAA
C203A F	CTGTAGGTAGAGCATTTGCTTTCATTGAAAAT
C203A R	GCAAATGCTCTACCTACAGTGACTATTCCAGC
C210A F	TCATTGAAAATCCCAAAGCTGAAGACTGTATC
C210A R	GCTTTGGGATTTTCAATGAAACAAAATGCTCT
C213A F	ATCCCAAATGTGAAGACGCTATCTTGAAGAAA
C213A R	GCGTCTTCACATTTGGGATTTTCAATGAAACA
C219A F	GTATCTTGAAGAAAGTAGCTAAATATTATAGA
C219A R	GCTACTTTCTTCAAGATACAGTCTTCACATTT

The substitution bases are underlined.

The italic bases represent restriction sites.

Table S2 Summary statistics of the predicted structure of Sis-HhH-GPD

All-Atom Contacts	Clashscore, all atoms:	9.3		75th percentile* (N=1784, all resolutions)
	Clashscore is the number of serious steric overlaps (> 0.4 Å) per 1000 atoms.			
Protein Geometry	Poor rotamers	0	0.00%	Goal: <0.3%
	Favored rotamers	207	99.52%	Goal: >98%
	Ramachandran outliers	0	0.00%	Goal: <0.05%
	Ramachandran favored	221	98.22%	Goal: >98%
	Rama distribution Z-score	-1.14 ± 0.50		Goal: abs(Z score) < 2
	MolProbity score^	1.49		95th percentile* (N=27675, 0Å - 99Å)
	Cβ deviations >0.25Å	0	0.00%	Goal: 0
	Bad bonds:	0 / 1890	0.00%	Goal: 0%
	Bad angles:	0 / 2540	0.00%	Goal: <0.1%
Peptide Omegas	Cis Prolines:	0 / 4	0.00%	Expected: ≤1 per chain, or ≤5%
Nucleic Acid Geometry	Bad bonds:	0 / 656	0.00%	Goal: 0%
	Bad angles:	1 / 1006	0.10%	Goal: <0.1%
Low-resolution Criteria	CaBLAM outliers	1	0.4%	Goal: <1.0%
	CA Geometry outliers	0	0.00%	Goal: <0.5%
Additional validations	Chiral volume outliers	0/399		
	Waters with clashes	0/0	0.00%	See UnDowser table for details

In the two colored column results, the left column gives the raw count and the right column gives the percentage.

*100th percentile is the best among structures of comparable resolution; 0th percentile is the worst. For clashscore the comparative set of structures was selected in 2004 and for MolProbity score in 2006.

^ MolProbity score combines the clashscore, rotamer, and Ramachandran evaluations into a single score, normalized to be on the same scale as X-ray resolution.

Table S3 Sequences of the oligonucleotides used to construct the *SiRe_0278* gene knockout

Name	Sequence (5′-3′)
Left F	ACGCGTCGACAAGGGTATTGTCGTGGGACCCATT
Left R	TCTATAATATTTATGTGTCAAGTATTTTACGAACCATT
Right F	AAATACTTGACACATAAATATTATAGAGAAAATTCCTCG
Right R	AAGGAAAAAAGCGGCCGCTCCTAACTCCCCTCTCTAGTGGTA
Spacer F	AACCAAACTTTCCACCAGCAGAGTACGGTAA
Spacer R	CTGGTGGAAAGTTTGGTTTGTGACCTGCGAA

The underlined bases represent restriction sites.

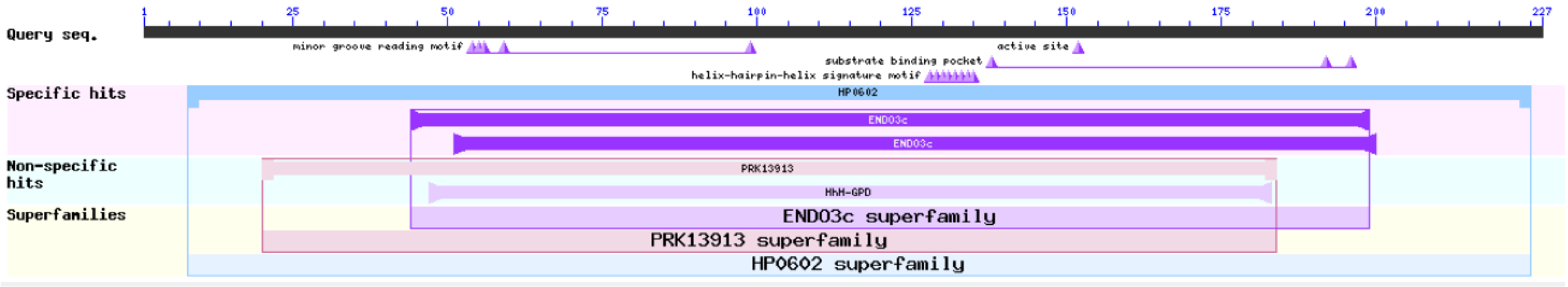

Fig. S1. Prediction of the conserved domains in Sis-HhH-GPD via conserved domain search.

ENDO3c superfamily (cd00056 and smart00478): endonuclease III (DNA-(apurinic or apyrimidinic site) lyase), alkylbase DNA glycosylases (AlkA-family) and other DNA glycosylases; PRK13913 superfamily (PRK13913): 3-methyladenine DNA glycosylase; HP0602 superfamily (COG2231): 3-methyladenine DNA glycosylase.

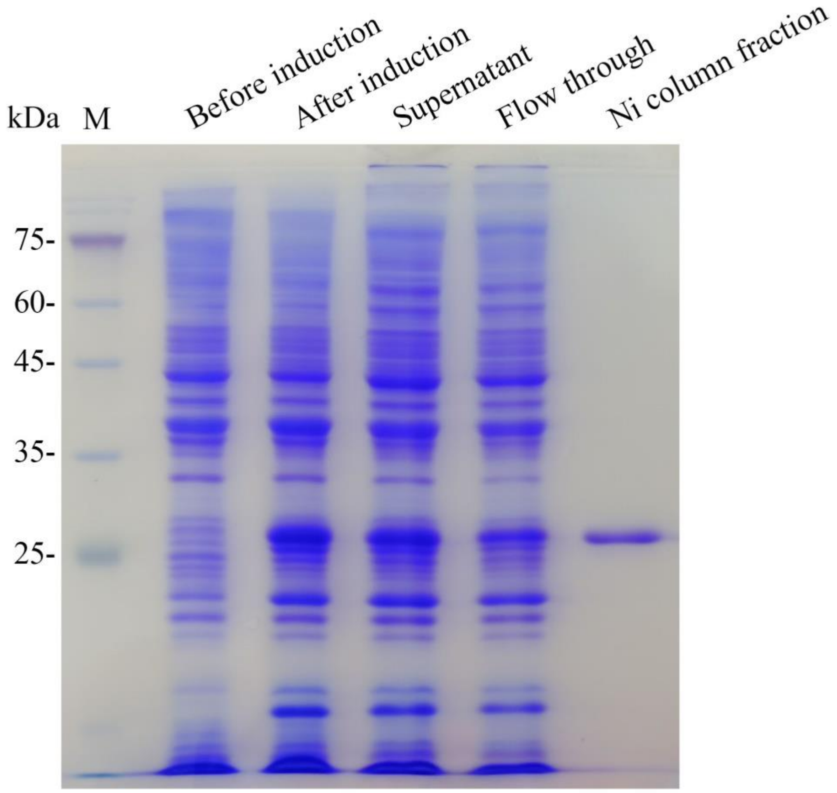

Fig. S2. Expression and purification of Sis-HhH-GPD protein. M: Marker.

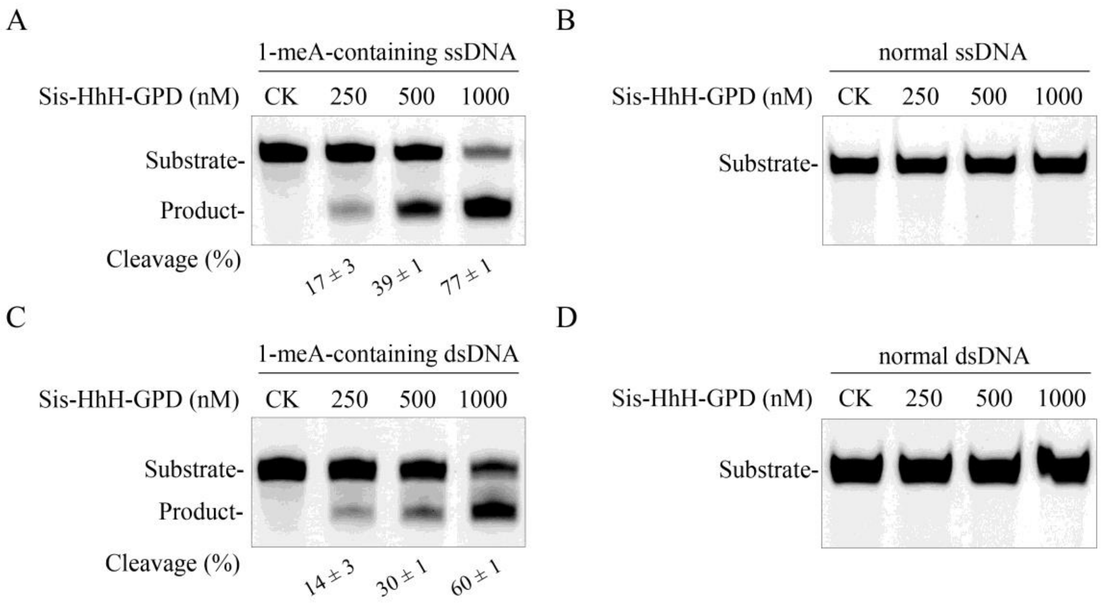

Fig. S3 Cleavage of 1-meA-containing DNA by Sis-HhH-GPD. 100 nM 1-meA-containing and normal DNA were used as substrates to perform DNA cleavage reactions of Sis-HhH-GPD with varied concentrations at 70 ℃ for 30 min. A. Cleavage of 1-meA-containing ssDNA by Sis-HhH-GPD. B. Cleavage of normal ssDNA by Sis-HhH-GPD. C. Cleavage of 1-meA-containing dsDNA by Sis-HhH-GPD. D. **C**leavage of normal dsDNA by Sis-HhH-GPD. CK: the reaction without Sis-HhH-GPD.

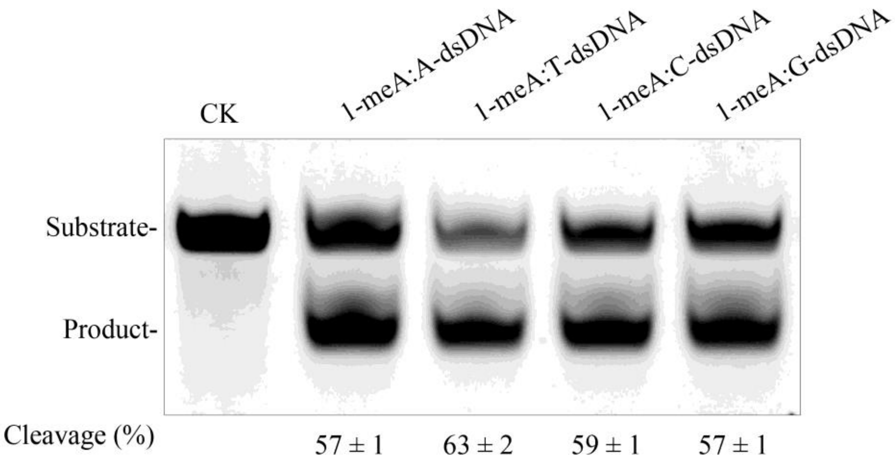

Fig. S4 Cleavage of 1-meA-containing dsDNA by Sis-HhH-GPD. 100 nM 1-meA-containing dsDNA substrate was used to perform DNA cleavage reactions in the presence of 1000 nM Sis-HhH-GPD at 70 ℃ for 30 min. CK: the binding without Sis-HhH-GPD.

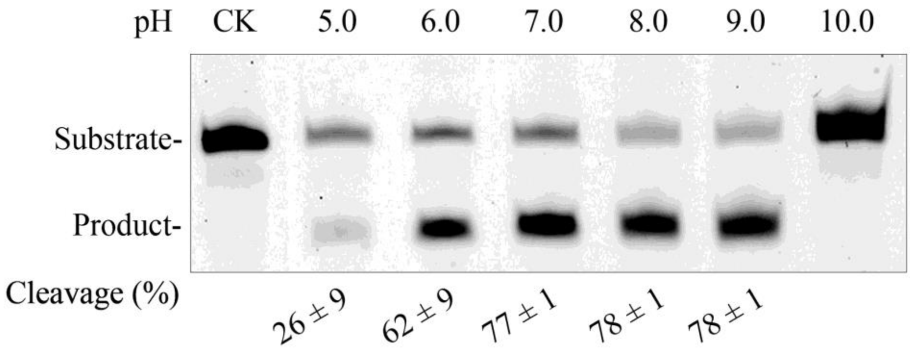

Fig. S5 The effect of pH on cleavage of 1-meA-containing DNA by Sis-HhH-GPD. 100 nM 1-meA-containing dsDNA substrate was used to perform DNA cleavage reactions of Sis-HhH-GPD at varied pHs at 70 ℃ for 30 min. CK: the binding without Sis-HhH-GPD.

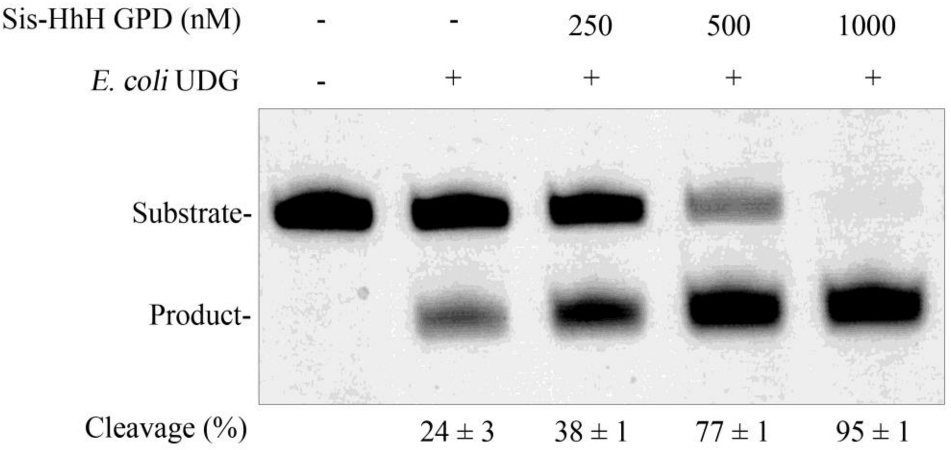

Fig. S6 Cleavage of AP-containing ssDNA by Sis-HhH-GPD. *E. coli* UDG is a mono-functional DNA glycosylase that can remove uracil from DNA. Thus, we incubated *E. coli* UDG with uracil-ssDNA at 37°C for 1 hr in the buffer (20 mM Tris–HCl pH 8.0, 1 mM DTT, and 8% glycerol) to prepared AP-containing ssDNA. The AP-containing ssDNA created by *E. coli* UDG was employed as substrate to perform DNA cleavage reactions of Sis-HhH-GPD with 250 nM, 500 nM and 1000 nM, respectively.

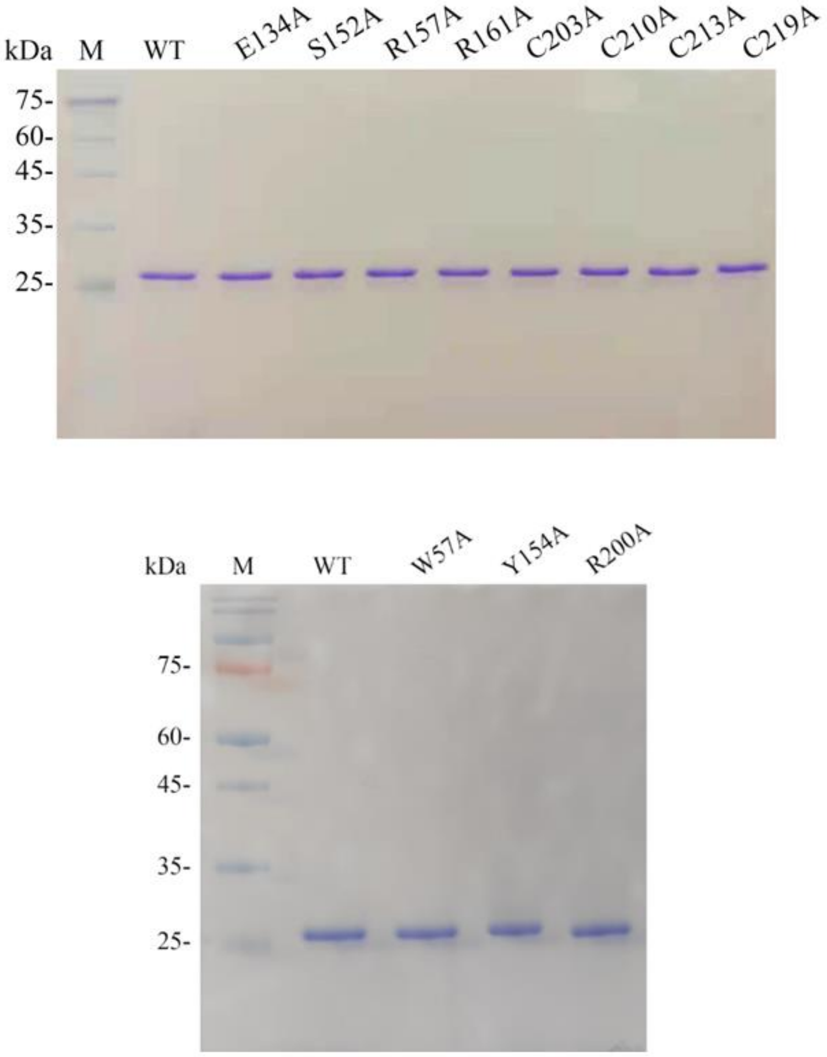

Fig. S7. Purification of the Sis-HhH-GPD mutant proteins. M: protein marker.

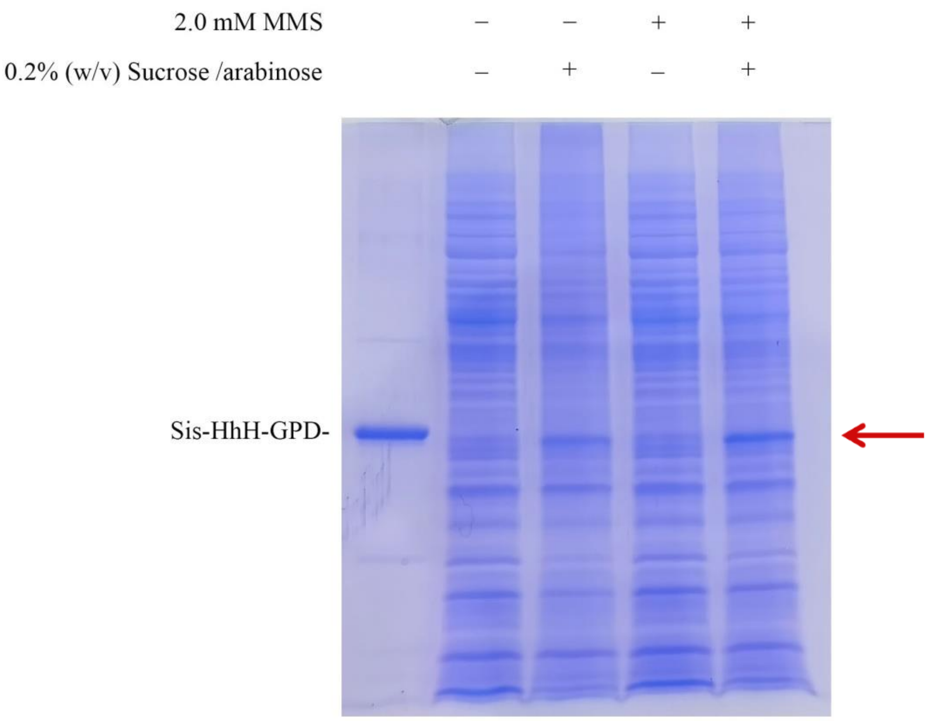

Fig. S8. The WT *SiRe_0278* gene was expressed in Δ*SiRe_0278* cells with the addition of 0.2% (w/v) sucrose and arabinose in the presence or absence of 2.0 mM MMS. The size of the purified His-tagged Sis-HhH-GPD protein from *E. coli* pLysS cells is larger than that of the expressed protein in *ΔSiRe_0278* strain since the former contains a longer vector sequence than the latter.

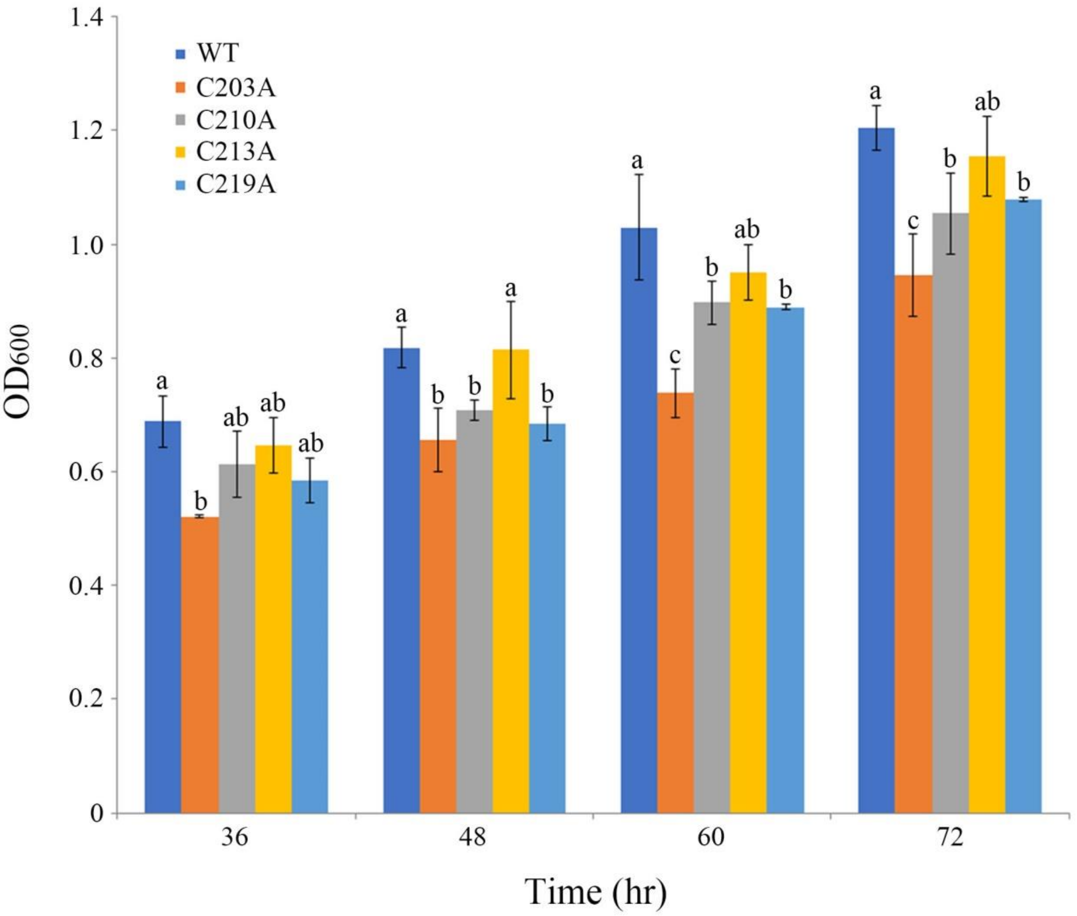

Fig. S9. Growth comparison of the WT and mutant recombinant Δ*SiRe_0278* cells with the addition of 0.2% (w/v) sucrose and arabinose in the absence of 2.0 mM MMS at 36, 48, 60, and 72 hr. Different letters indicate significant difference at the 5% level

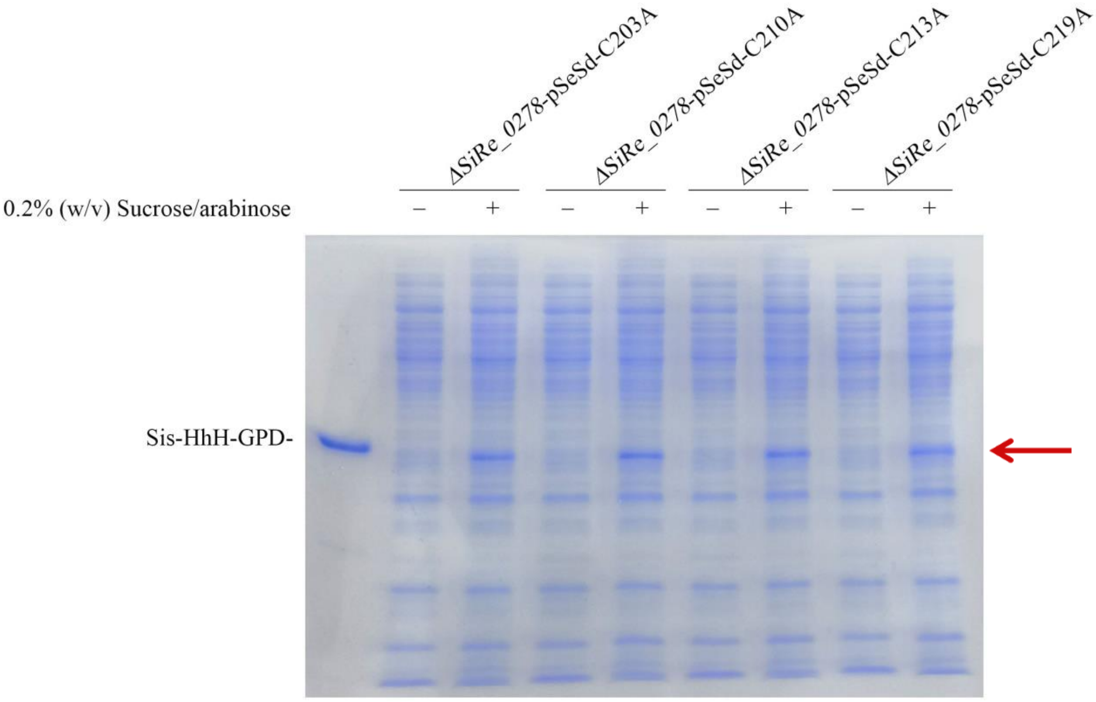

Fig. S10. The mutant *SiRe_0278* genes were expressed in Δ*SiRe_0278* cells with the addition of 0.2% (w/v) sucrose and arabinose in the presence of 2.0 mM MMS.
